# HalALMT1 mediates malate efflux in the cortex of mature cluster rootlets of *Hakea laurina*, occurring naturally in severely phosphorus‐impoverished soil

**DOI:** 10.1111/nph.70010

**Published:** 2025-02-24

**Authors:** Hirotsuna Yamada, Lydia Ratna Bunthara, Akira Tanaka, Takuro Kohama, Hayato Maruyama, Wakana Tanaka, Sho Nishida, Tantriani, Akira Oikawa, Keitaro Tawaraya, Toshihiro Watanabe, Shu Tong Liu, Patrick M. Finnegan, Hans Lambers, Takayuki Sasaki, Jun Wasaki

**Affiliations:** ^1^ Graduate School of Integrated Sciences for Life Hiroshima University Higashi‐Hiroshima Hiroshima 739‐8521 Japan; ^2^ School of Biological Sciences University of Western Australia Perth 6009 WA Australia; ^3^ Faculty of Agriculture Saga University Saga Saga 840‐0027 Japan; ^4^ School of Integrated Arts and Sciences Hiroshima University Higashi‐Hiroshima Hiroshima 739‐8521 Japan; ^5^ School of Agriculture Hokkaido University Sapporo Hokkaido 060‐0809 Japan; ^6^ The United Graduate School of Agricultural Sciences Kagoshima Kagoshima 890‐0064 Japan; ^7^ Faculty of Agriculture Yamagata University Tsuruoka Yamagata 997‐8555 Japan; ^8^ Faculty of Agriculture Universitas Gadjah Mada Yogyakarta 55281 Indonesia; ^9^ Graduate School of Agriculture Kyoto University Kyoto Kyoto 606‐8502 Japan; ^10^ Agriculture Green Academy China Agricultural University Beijing 100083 China; ^11^ Beijing Forestry University Beijing 100083 China; ^12^ Institute of Plant Science and Resources Okayama University Kurashiki Okayama 710‐0046 Japan; ^13^ Seto Inland Sea Carbon Neutral Research Center Higashi‐Hiroshima Hiroshima 739‐8521 Japan

**Keywords:** aluminum‐activated malate transporter, cluster root, cortex, *Hakea laurina*, phosphorus, Proteaceae

## Abstract

- *Hakea laurina*, a woody Proteaceae, naturally occurs in severely phosphorus (P)‐impoverished habitats in southwest Australia. It develops distinctive cluster roots that exhibit a high capacity for carboxylate exudation and acid phosphatase activity, contributing to its P acquisition. However, the molecular mechanisms underlying these physiological functions remain poorly understood.
- We explored the cluster‐root transcriptome using *de novo* RNA‐Seq and identified *Hakea laurina Aluminum‐activated Malate Transporter 1* (*HalALMT1*), encoding an aluminum (Al)‐activated malate transporter induced in mature cluster roots. We characterized HalALMT1 through electrophysiological assays and overexpression in *Arabidopsis thaliana*, and localized *HalALMT1* expression, acid phosphatase activity, and suberized boundaries in cluster roots.
- Differentially expressed genes highlighted multiple increased carboxylate‐related processes at cluster‐root maturity. HalALMT1 released malate, an activity further enhanced by exposure to Al^3+^. Notably, *HalALMT1* was specifically expressed in mature cortex cells of cluster rootlets, which lack a suberized exodermis. Acid phosphatase activity was pronounced throughout the cluster rootlets, unlike in noncluster roots where it was limited to the epidermis and stele.
- Substantial malate release and acid phosphatase activity in the cortex cells in cluster rootlets, which lack a suberized exodermis, allowed massive exudation. This study sheds light on an exquisite P‐acquisition strategy of Proteaceae, enabling survival under extremely low P availability.

## Introduction

Plants in extremely phosphorus (P)‐impoverished habitats, such as southwest Australia, possess highly efficient P‐acquisition strategies. For example, most Proteaceae, one of the dominant plant families in southwest Australia, form cluster roots under low‐P conditions, characterized by a large number of determinate rootlets developing from a short region of the root axis (Shane & Lambers, 2005). Cluster roots are short‐lived, typically lasting *c*. 3 wk, and develop periodically along the root axis (Shane & Lambers, 2005). Cluster roots from most plants that express them release massive amounts of carboxylates and acid phosphatase at maturity. Acid phosphatase hydrolyzes organic P (Shane & Lambers, 2005), which is important since plants only take up inorganic phosphate at physiological pH, primarily as H_2_PO_4_^−^ (Lambers, 2022). The carboxylates released are mainly citrate and malate, which bind to P‐sorption sites on soil particles, displacing P (Roelofs *et al*., 2001). Shane *et al*. (2004a) reported that *Hakea prostrata* (Proteaceae) exhibits a burst of carboxylate exudation from mature cluster roots when the rootlets reach their final length at *c*. 12 d after emergence. Citrate and malate exudation are particularly dominant. The release of protons (H^+^) provides the driving force for carboxylate release and act as counterions to balance the negative charge of the released carboxylates, thereby acidifying the rhizosphere (Shane & Lambers, 2005; Lambers *et al*., 2018).

Aluminum (Al) released from soil minerals into the soil solution under acidic conditions is mainly in the form Al(H_2_O)_6_^3+^ (referred to hereafter as Al^3+^), although there are also mononuclear hydrolysis products such as Al(OH)^2+^, Al(OH)_2_^+^, Al(OH)_3_, and Al(OH)_4_^−^ in the soil, depending on soil pH (Rengel, 2023). Trivalent Al^3+^ is toxic, making it a significant factor limiting plant productivity in acid soils (Kochian *et al*., 2004). While P sorption due to Al minerals is predominant at pH 5–6, the solubility of Al increases as the pH decreases and P sorption shifts to Fe minerals (Lindsay, 1979). Aluminum toxicity is particularly pronounced below pH 5 (Weber & Peuker, 2020). Aluminum ions can be detoxified through chelation by carboxylates such as malate and citrate, which plants, particularly Proteaceae, exude from their roots (Álvarez‐Fernández *et al*., 2014). Thus, carboxylate exudation contributes to both P mobilization and Al detoxification (Delhaize *et al*., 1993; Lambers *et al*., 2018), which emphasizes the importance of carboxylate exudation as an adaptive mechanism to cope with both low P availability and Al toxicity.

An aluminum‐activated malate transporter (ALMT) is responsible for malate exudation from roots. Since the first isolation of *ALMT1* in *Triticum aestivum* (Sasaki *et al*., 2004), it was established that it belongs to a highly conserved gene family in plants, promoting Al detoxification in *Arabidopsis thaliana* (Hoekenga *et al*., 2006; Kobayashi *et al*., 2007), *Brassica napus* (Ligaba *et al*., 2006), and *Holcus lanatus* (Chen *et al*., 2013). The availability of carboxylate transporters comprises the rate‐limiting step for Al‐activated carboxylate exudation from roots, rather than carboxylate concentrations in the cytosol (Liu *et al*., 2009). *ALMT* genes have multiple functions. For example, *Glycine max GmALMT5* (Peng *et al*., 2018), *Arabidopsis thaliana AtALMT3* (Maruyama *et al*., 2019), and *Lupinus albus LaALMT1* (Zhou *et al*., 2020) are involved in P acquisition in roots, while *AtALMT4* (Eisenach *et al*., 2017), *AtALMT6* (Meyer *et al*., 2011; Ye *et al*., 2021), *AtALMT9* (De Angeli *et al*., 2013), and *AtALMT12* (Meyer *et al*., 2010; Sasaki *et al*., 2010) are involved in stomatal movement in *A. thaliana*.

The molecular aspects of cluster‐root functioning and carboxylate exudation have yet to be investigated in a P‐efficient Proteaceae. This may be due to the challenges of nucleic acid extraction from woody species. They also exhibit extremely efficient P remobilization during senescence and low levels of RNA (Denton *et al*., 2007; Bird *et al*., 2024a). By contrast, several RNA‐Seq analyses have been carried out in *Lupinus albus* (Fabaceae), a legume crop that also forms clusters roots (Secco *et al*., 2014; Wang *et al*., 2014; Le Thanh *et al*., 2021). While cluster roots of *L. albus* are much smaller with less densely packed rootlets than those of most Proteaceae, their functioning is similar. These studies have provided transcriptomic insights into the physiological activities of cluster roots related to P acquisition based on the accumulation of transcripts associated with phosphate transporters, carboxylate transporters, and acid phosphatases in cluster roots (Secco *et al*., 2014; Wang *et al*., 2014; Le Thanh *et al*., 2021). This study represents the first exploration of how cluster roots of Proteaceae respond to low‐P conditions at the transcript level.

*Hakea laurina* (Proteaceae) endemic to a restricted area of southwest Australia (Fernandes *et al*., 2022) and forms cluster roots in low‐P environments (Lamont, 2003). *H. laurina* is expected to have evolved an advanced strategy to tolerate low P availability, especially with respect to molecular biological characteristics, that is distinct from crops. In this study, using *H. laurina* as an example of a cluster‐rooted Proteaceae, we aimed to explore how it survives in an extremely P‐limited environment through understanding its root physiology, the gene expression patterns that underpin those physiological traits, and the function of ALMT in mature cluster roots. We focused on four topics: (1) fieldwork and hydroponic growth experiments to assess its response to P; (2) investigations on physiological functions for P acquisition in cluster roots; (3) transcriptome analysis focusing on P‐starvation‐responsive genes induced in mature cluster roots; and (4) isolation and functional analysis of an *ALMT* transcript induced in mature cluster roots under low P conditions.

## Materials and Methods

### Study site, sample collection, and analysis

Fieldwork for this study was conducted at four sites within Fitzgerald River National Park, which has a Mediterranean climate (Gentilli, 1972). The mean annual rainfall, mean monthly maximum temperature in January (summer), and minimum temperature in July (winter) at Jacup (33.89°S, 119.11°E), close to the target sites, are 452.4 mm, 27.9°C, and 15.8°C, respectively (Australian Bureau of Meteorology, http://www.bom.gov.au/climate/data/). The coordinates for Site1, Site2, Site3, and Site4 are 34°11′57.0″S 119°21′36.3″E, 34°12′47.4″S 119°26′08.9″E, 34°10′36.0″S 119°33′39.9″E, and 34°10′07.″S 119°30′35.5″E, respectively (Supporting Information Methods S1). Mature fully expanded leaves of *H. laurina* R. Br. and bulk soil were collected from three mature plants at each site. The leaves were wiped with tissue to remove soil dust, oven‐dried at 60°C for 3 d, and then ground to fine powder using a vertical ball‐mill grinder (Geno/Grinder 2010; Spex, SamplePrep, Metuchen, NJ, USA) with plastic 5‐ml tubes and yttrium‐stabilized zirconium ceramic beads. The ground samples were used to determine the concentration of P, Al, and manganese (Mn) in the leaves. For P determination, 50 mg of ground samples was digested in H_2_SO_4_–H_2_O_2_, and the P concentration in the solution was determined using a molybdenum blue method (Murphy & Riley, 1962). Another 50 mg of ground samples was digested in 2 ml of 60% (v/v) HNO_3_. The digested samples were diluted with 10 ml of 2% (v/v) HNO_3_ and analyzed for Al and Mn using inductively coupled plasma mass spectrometry (ICP‐MS; NexION 2000C; PerkinElmer, Waltham, MA, USA). The validity of the analytical method for elements, including aluminum, in plant samples was confirmed using standard reference materials (NCS DC73349, China National Analysis Center for Iron and Steel; BCR‐129, Joint Research Centre, European Commission).

Soil was air‐dried at room temperature for 7 d and sieved (< 2 mm). Soil pH was measured using a pH probe (Orion 720a; Beverly, MA, USA) calibrated with pH 4 and 7 buffer solutions, with a soil to solution ratio of 1 : 5 in water. The total P was determined by igniting the soil at 550°C for 1 h followed by extraction with HCl in a 1 : 30 soil to 1 M HCl ratio, with shaking for 16 h. Inorganic P was determined by extracting the soil with HCl in a 1 : 10 soil to 1 M HCl ratio, with shaking for 16 h. For resin P, the soil was extracted using 30 ml of Milli‐Q water with four anion‐exchange membranes, after which the membranes were immersed in 10 ml of 0.5 M HCl and shaken for 1 h. The P concentration was determined using a malachite green‐based assay (Motomizu *et al*., 1983) with a UV–VIS spectrophotometer (UV160A; Shimadzu, Kyoto, Japan) at 630 nm.

### Plant growth

Seeds of *H. laurina* were germinated in vermiculite for 40 d. Seedlings were then transferred onto a floating platform and hydroponically cultured in a container filled with 4 l of nutrient solution with six plants per container. The nutrient solution composition was 2.1 mM NH_4_NO_3_, 0.77 mM K_2_SO_4_, 1.2 mM CaCl_2_, 0.82 mM MgSO_4_, 36 μM Fe‐ethylenediaminetetraacetic acid, 9.1 μM MnSO_4_, 46 μM H_3_BO_3_, 3.1 μM ZnSO_4_, 0.16 μM CuSO_4_, 0.052 μM (NH_4_)_6_Mo_7_O_24_ with no added P. After 32 d of acclimation in the 0 μM P hydroponic solution, three containers of seedlings were subjected to one of three P treatments (0, 6.4, and 64 μM P) in the above nutrient solution for 43 d. The initial pH of the nutrient solution was adjusted to 5.6–5.8, and continuous aeration was provided. The containers were covered with black tape to shield the belowground part of the seedlings from light. The nutrient solution was replaced weekly. Plants were grown on a shelf near the window to receive natural sun light at *c*. 25°C.

After 43 d of P treatments, three plants from each of the three containers for each treatment were harvested for analysis. Plants were *c*. 4 months of age. Shoots and roots were collected separately. All samples were dried at 70°C for 3 d after measuring fresh weight and counting the number of cluster roots. Dried mature leaves were used to determine the P concentration using the molybdenum blue method (Murphy & Riley, 1962).

The remaining three plants in each container of the P treatments were transferred to a glasshouse and hydroponically cultivated in nutrient solution that was supplemented with 6.4 μM NaH_2_PO_4_ every 2–3 months to encourage growth of cluster roots.

After 27 months, the above seedlings were used to measure carboxylate‐exudation rate and the activity of acid phosphatase. Developing cluster roots were divided into four groups: tip of cluster‐bearing lateral roots (TCR), young cluster roots (YCR), white mature cluster roots (MCR) with fully elongated rootlets, and senesced cluster roots (SCR) with a dark‐brown color. Physiologically active roots were screened by incubating them on agar containing 0.008% (w/v) bromocresol purple, a pH indicator, for 30 min. The four developmental stages of cluster roots from each plant were submerged in 5 ml of deionized water for 3 h to collect root exudates. Exudates were immediately filtered using a MILLEX GV Filter Unit (0.45 μm; Millipore, Burlington, MA, USA) for analysis of root exudates.

Another batch of seedlings was germinated and transferred to hydroponic culture supplemented with 6.4 μM NaH_2_PO_4_ every 2–3 months in a glasshouse as described previously. Mature cluster roots and tips of cluster‐bearing lateral roots that were produced when these plants were *c*. 3 yr old were harvested. Roots were promptly frozen in liquid nitrogen and stored at −80°C for RNA extraction. After other mature cluster roots were exposed to 100 μM AlCl_3_ for 3 h, they were also harvested, frozen, and stored as described previously for quantitative polymerase chain reaction analysis. Mature leaves from these 3‐yr‐old seedlings were collected and dried to determine P concentration as descried previously.

Plants from the second batch of seedlings were further grown in hydroponics supplemented with 6.4 μM NaH_2_PO_4_ every 2–3 months. Mature cluster roots were harvested when these plants were *c*. 5 yr old for *in situ* hybridization, histochemical analysis of acid phosphatase activity, and observation of suberized lamellae. Mature leavers from these plants were also collected and dried to determine P concentration as described previously.

### Physiological analysis

The concentrations of malate and citrate in root exudates were determined using enzyme assay kits for malic acid and citric acid (F‐kit; Roche Diagnostics, Basel, Switzerland) and a spectrophotometer (Multiskan GO; Thermo Fisher Scientific) at 340 nm. Exudation rates were calculated based on root fresh weight and the collection period. Acid phosphatase activities were determined following the method of Wasaki *et al*. (2005). Briefly, activity was measured using the fluorescent substrate 4‐methylumbelliferyl phosphate. Enzyme activities were quantified by calculating the reaction rate based on the calibration curve created with methylumbelliferone. All measurements were conducted using a multiplate reader (2030 ARVO X; PerkinElmer, Waltham, MA, USA). The remaining exudate samples were freeze‐dried for capillary‐electrophoresis time‐of‐flight/Mass spectrometry (CE‐TOF/MS) analysis. All CE/TOF MS experiments were performed according to Tawaraya *et al*. (2014) using a capillary electrophoresis system (Agilent Technologies, Santa Clara, CA, USA). The relative molar concentration of metabolites in root exudates was calculated using the concentration of malate in mature cluster‐root exudates as the standard (standard value = 100).

### RNA‐Seq analysis

The total RNA from frozen roots was extracted (RNeasy Plant Mini Kit; Qiagen) with modifications for woody plant tissues. Briefly, 50 mg frozen sample was homogenized in 1 ml extraction buffer containing 4 M guanidine isothiocyanate, 0.2 M sodium acetate at pH 5.0, 25 mM EDTA, 2.5% (w/v) polyvinylpyrrolidone‐25 (PVP‐25), and 1% (v/v) β‐mercaptoethanol. After homogenization, 100 μl of 20% (w/v) sarkosyl was added, and the homogenates were incubated at 70°C for 10 min with occasional mixing by vortex. All homogenates were processed using QIAshredder Spin Columns, as specified by the manufacturer. The quality and quantity of extracted RNA were verified using a microphotometer (NanoDrop; Thermo Fisher Scientific). Aliquots of the RNA samples were used for RNA‐Seq analysis after ensuring the RNA quality by microchip electrophoresis (MultiNA; Shimazu Corp., Kyoto, Japan).

Identification of differentially expressed genes was conducted following the workflow summarized in Methods S2. The cDNA libraries for RNA‐Seq were prepared (NEBNext Ultra ll Directional RNA Library Prep Kit for Illumina, NEBNext Multiplex Oligos for Illumina, and NEBNext Poly(A) mRNA Magnetic Isolation Module; New England Biolabs Japan, Inc., Tokyo, Japan), and subsequently sequenced as 150 bp paired‐end reads (HiSeqX; Macrogen Japan Corp., Tokyo, Japan) after being qualified and quantified (NEBNext Library Quant Kit for Illumina, MultiNA; Shimazu Corp.). Raw data were cleaned by removing adapter sequences and low‐quality sequences (Trimmomatic; v.0.39; http://www.usadellab.org/cms/?page=trimmomatic). The cleaned RNA‐Seq reads were assembled (Trinity, v.2.15.1; https://github.com/trinityrnaseq/trinityrnaseq/releases/tag/Trinity‐v2.15.1) to obtain a reference *de novo* transcriptome of *H. laurina*. The expression levels of contigs were quantified (Salmon, v.1.9.0; https://github.com/COMBINE‐lab/salmon) and expressed as counts per million (CPM). Contigs with low signal (CPM < 0.4) were removed (Nishida *et al*., 2017). Expression data were normalized across the samples, and 0.01 CPM was added to eliminate zero values. All normalized data were used to calculate the fold‐change of gene expression between tips of cluster‐bearing lateral roots and mature cluster roots (edgeR, v.4.0.14; https://bioconductor.org/packages/release/bioc/html/edgeR.html). Putative open reading frames and peptide sequences encoded by the assembled transcripts were predicted (TransDecoder, v.5.7.1; https://hpc.nih.gov/apps/TransDecoder.html). A total of 7819 annotated genes were considered to be differentially expressed genes (DEGs) through pairwise comparison using edgeR, with a *q*‐value < 0.05 and |log_2_ fold‐change| ≥ 1. Gene ontology (GO) and Kyoto Encyclopedia of Genes and Genomes (KEGG) pathway analysis were performed (ShinGO 0.80, http://bioinformatics.sdstate.edu/go/) with a false discovery rate (FDR) cutoff of 0.05. The Welch *t*‐test (*P* < 0.05, *n* = 3) was also applied to 4210 DEGs upregulated genes in mature cluster roots to select candidate genes. A heatmap (log_10_CPM) was used to visualize the expression of these genes (ggplot2, v.3.5.1; https://ggplot2.tidyverse.org/). Phylogenetic analysis was carried out using ClustalW (Thompson *et al*., 1994) and Mega11 software (https://www.megasoftware.net/). Gene expression was confirmed using 100 ng of total RNA in quantitative polymerase chain reaction analysis as described by Yamada *et al*. (2022). An *H. laurina* ortholog (*HalEF1a*) encoding elongation factor 1A in *A. thaliana* (AT1G07940) was used as a housekeeping gene. Primer pairs are summarized in Table S1.

### Cloning *of HalALMT1* and its promoter sequence

The coding region of *Hakea laurina Aluminum‐activated Malate Transporter 1* (*HalALMT1*) (accession no.: LC833900) was PCR‐amplified from *H. laurina* cDNA with primers (Table S2) and subcloned into the *Bgl* II site of the oocyte expression vector pXBG2 (Sasaki *et al*., 2014) via the In‐Fusion cloning technique (In‐Fusion HD cloning kit; Takara‐bio, Shiga, Japan) for electrophysiological characterization. Full‐length HalALMT1 and HalALMT1 without a stop codon were cloned into the entry vector pCR™8/GW/TOPO (Thermo Fisher Scientific) using the TA cloning technique with primers shown in Table S2. The cloned sequences were confirmed using inner primers (Table S2). The confirmed full‐length HalALMT1 sequence was transferred into the Gateway destination vector pGWB2 and pGWB6 for N‐terminal green fluorescent protein (GFP) fusion, and HalALMT1 without a stop codon was transferred into the Gateway destination vector pGWB5 for C‐terminal GFP fusion (Nakagawa *et al*., 2007) via LR recombination. Finally, HalALMT1::GFP (C‐terminal fusion) and GFP::HalALMT1 (N‐terminal fusion) amplified from *HalALMT1* in pGWB5 and pGWB6 using primers (Table S2) were cloned into the *Bgl* II site of pXBG2 using In‐Fusion HD cloning kit for electrophysiological characterization.

Genomic DNA of *H. laurina* was extracted from leaves following the method described by Tapia‐Tussell *et al*. (2006). The DNA was digested with *Sph1* and self‐ligated (Ligation high, Toyobo, Osaka, Japan). An *c*. 4.5 kbp fragment encompassing *HalALMT1* promoter was obtained via nested PCR using two primer sets (Table S2). After sequencing by the primer walking, a 2862 bp region upstream of *HalALMT1*, amplified by PCR, was subcloned into the pCR™‐Blunt2‐TOPO vector (Thermo Fisher Scientific) for promoter analysis. The primers used are listed in Table S2.

### Electrophysiological measurements

Electrophysiological measurements and fluorescence images in *Xenopus* oocytes were performed using the methods described previously (Furuichi *et al*., 2010; Sasaki *et al*., 2014). Briefly, cRNA of HalALMT1 were synthesized and injected into *Xenopus* oocytes. cRNA‐injected and water‐injected oocytes as a control were injected with 50 μl of 0.1 M malate treated for RNA. After the incubation for 3 or 4 d, two‐electrode voltage clamp analysis was conducted as described previously (Sasaki *et al*., 2014). The oocyte membrane was clamped to a holding potential of −20 mV, and voltage pulses were applied in 20 mV increments from −140 to +40 mV (Axoclamp 900A with the pClamp 10 software; Molecular Devices, San Jose, CA, USA). The bath solutions consisted of ND96 (96 mM NaCl, 1.8 mM CaCl_2_, 1 mM KCl, 0.1 mM LaCl_3_, pH 4.5, adjusted to 210 mOsm kg^−1^ with sorbitol). To assess the activation of HalALMT1 activity by Al^3+^, 100 μM AlCl_3_ in ND95 solution was used. Oocytes expressing HalALMT1::GFP (C‐terminal fusion) were used to determine intracellular localization in oocytes. They were fixed with formaldehyde, embedded in agarose, and sliced into 50‐μm‐thick sections. Fluorescence images from sectioned oocytes were observed with a confocal laser microscope (LSM510; Carl Zeiss Inc., Oberkochen, Germany) with excitation at 488 nm and a 505–530 nm bandpass filter.

### Overexpression of HalALMT1 in *Arabidopsis thaliana*

A loss‐of‐function mutant for AtALMT1 in *A. thaliana* (*atalmt1*; SALK_009629) was obtained from the *Arabidopsis* Biological Resource Center. *A. thaliana* Col‐0 wild‐type (WT) was used as the control. For transfection, pGWB2‐*HalALMT1* and pGWB6‐*HalALMT1* were introduced into *Agrobacterium tumefaciens* strain EHA101 (Hood *et al*., 1986) or LBA4404 (Takara‐bio). Transgenics of both *atalmt1* and Col‐0 *A. thaliana* were generated by floral dipping (Clough & Bent, 1998) using *A. tumefaciens* harboring pGWB2‐*HalALMT1* or pGWB6‐*HalALMT1*. Transgenics (T1) were selected by germinating seeds on ½ Murashige and Skoog (MS) medium containing 20 μg ml^−1^ hygromycin,100 μg ml^−1^ carbenicillin, and 0.8% (w/v) agar (pH 5.7). One‐week‐old seedlings were transferred to ½ MS medium supplemented with 100 μg ml^−1^ carbenicillin and 0.8% (w/v) agar (pH 5.7) for an additional week, after which they were transplanted to soil. More than 2000 seeds harvested from three T1 plants were sown on ½ MS medium containing 20 μg ml^−1^ hygromycin and 0.8% (w/v) agar (pH 5.7) to select T2 plants, which were used for determining malate‐exudation rates or GFP fluorescence.

*Arabidopsis thaliana* seeds were surface‐sterilized with 10% (v/v) sodium hypochlorite and germinated on ½ MS medium containing 0.8% (w/v) agar (pH 5.7) for 1 wk. Seedlings were transferred to ½ MS medium containing 0.5% (w/v) phytagel (pH 5.7). Col‐0 and transgenic 35Spro‐*GFP*::*HalALMT1*/Col‐0 plants were grown in a growth chamber under 16 h : 8 h, 25°C : 20°C, light : dark (220 μmol m^−2^ s^−1^). Twenty‐day‐old seedlings were used for GFP observation. Before observation, roots were stained with 20 μM FM4‐64 (Thermo Fisher Scientific) for 5 min to visualize the plasma membrane. After washing three times with distilled water, roots were placed in a glass‐bottom dish (Matsunami Glass Ind., Ltd, Osaka, Japa) and imaged using a confocal laser microscope (FV3000; Olympus, Tokyo, Japan). GFP fluorescence was observed using 488 nm excitation and 505–530 nm emissions wavelengths, while FM4‐64 staining was observed with a > 560 nm bandpass filter. Images were processed using the Fiji software (https://fiji.sc/).

Fifteen 25‐d‐old seedlings of WT and two transgenic lines of 35Spro‐*HalALMT1*/Col‐0 in triplicate were transferred to 10 ml of distilled water or 10 ml 13.6 μM AlCl_3_ solution for 24 h to collect root exudates. Roots from each line were immediately frozen in liquid nitrogen and stored at −80°C for further analysis. The exudates were immediately filtered using a MILLEX GV Filter Unit (0.45 μm; Millipore). Malate concentrations in the solution were determined using ion chromatography (Eco IC; Metrohm, Herisau, Switzerland), and the rates of malate exudation were calculated based on root fresh weight and incubation time. Total RNA from frozen samples was extracted (Plant Total RNA Extraction Mini Kit; Favorgen Biotech, Ping‐Tung, Taiwan). An aliquot containing 500 ng total RNA was used for quantitative polymerase chain reaction analysis using *HalEF1a* and *HalALMT1* primers (Table S1) to determine gene expression levels.

Fifteen 10‐d‐old seedlings of Col‐0, *atalmt1*, and 35Spro‐*HalALMT1*/*atalmt1 A. thaliana* were transferred to ½ MS plates containing 0.5% (w/v) phytagel (pH 4.8) without P in the presence or absence of 300 μM AlCl_3_ for 7 d. The lengths of taproots from the top five seedlings were measured, and the average value was calculated for each replicate. Relative root length under Al condition (%) was calculated as (root length under + AlCl_3_)/(root length under − AlCl_3_)*100. Roots from each line were immediately frozen in liquid nitrogen and stored at −80°C until analysis. The frozen samples were used for quantitative polymerase chain reaction using *HalEF1a*, *AtALMT1*, and *HalALMT1* primers (Table S1) to determine gene expression levels.

### *In situ* hybridization

The deduced HalALMT1 amino acid sequence was used in a Blastp (v.2.12.0) search against all putative open reading frames identified by RNA‐Seq to identify the top five HalALMT1‐homologous transcripts containing a 3′‐UTR sequence. These transcript sequences were aligned using Clastal W (Thompson *et al*., 1994) to select a probe site for the specific detection of *HalALMT1* (Methods S3). A 540 bp cDNA fragment, including 3′‐UTR, that had less than 60% identity with homologous *HalALMT* transcripts was amplified using primers (Table S2) and cloned into pBluescript ll SK (+). RNA was transcribed with T7 RNA polymerase using digoxigenin‐labeled UTPs (Roche) to generate an antisense probe.

Cluster roots from the same batch of low‐P plants used for RNA‐Seq analysis were harvested when the plants were *c*. 5 yr old. Cluster roots were fixed and dehydrated following the methods of Itoh *et al*. (2000), and subsequently impregnated with xylene before embedding (Paraplast Plus; Oxford Labware, St. Louis, MO, USA). Microtome sections (8 μm) were mounted on glass slides. *In situ* hybridization and immunological detection of signals were performed according to Toriba & Hirano (2018). The optimal amounts of probe, hybridization period, and coloring period for observation were 4 μl, 20 h, and 3 h, respectively. Samples were observed (BX53LED optical microscope; Olympus) and imaged (Axiocam 506 color camera; Carl Zeiss).

### Activity staining using fluorescent substrate

Acid phosphatase activity was visualized using the method of Wasaki *et al*. (2008), with minor modifications. Briefly, cluster roots from the same batch of low‐P plants used for RNA‐Seq analysis were harvested when the plants were *c*. 5 yr old. Root samples were washed and embedded in optimum cutting temperature (OCT) compound and sliced into 50‐μm sections (Cryostat CM3050S; Leica, Solms, Germany) following the protocol of Kobayashi *et al*. (2021). Cross sections were stretched onto a slide glass (CRE‐05 Green; Matsunami Glass Ind., Ltd) for observation of acid phosphatase activities and suberin layers. ELF™ 97 phosphate (Invitrogen) was used as a substrate for acid phosphatase (Nedoma *et al*., 2003; Nedoma & Vrba, 2006) for histochemical activity staining. A 0.1 M 2‐morpholinoethanesulfonic acid (MES) buffer adjusted to pH 5.5 was used for substrate dilution and washing. ELF97 phosphate was diluted 40 times with 0.1 M MES, pH 5.5. Aliquots of 500 μl diluted substrate were applied to each slide glass, which were then incubated in a dark room at room temperature for 15 min. After the incubation, the substrate was removed, and slides washed three times with 0.1 M MES, pH 5.5 to eliminate residual substrate. Wash buffer (50 μl) was applied to the washed root sections, the sections were covered with a cover glass, and the ELF97 signal was visualized using a fluorescence microscope (APEXVIEW APX100; Olympus) equipped with U‐FUNA filter sets: excitation 360–370 nm, dichroic mirror 410 nm, and emission 420–460 nm. The color of the images was measured using the Fiji software (https://fiji.sc/).

### Histochemical detection of suberin in cluster roots of *Hakea laurina*

Cross sections of cluster roots were obtained as described previously for acid phosphatase activity. Fluorol Yellow 088 (ChemScene, Monmouth Junction, NJ, USA) was used for suberin staining. A 500 μl aliquot of 0.01% (v/w) Fluorol Yellow 088 was applied to each slide and incubated for 16 h in a dark room at room temperature. After incubation, the solution was removed, and the slides were washed three times with water. Subsequently, 500 μl of CitiFluor™ AF1 Mountant Solution (PST proSciTech, Thuringowa, Australia) was added and slides incubated for 1 h at room temperature. After mounting, 50 μl of 75% (v/v) glycerol was applied to the washed root sections, which were then covered with a cover glass. The suberin signal was detected using a fluorescence microscope (APEXVIEW APX100; Olympus) with U‐FBNA filter sets: excitation at 488 nm, a dichroic mirror at 505 nm, and emission at 510–550 nm.

### Statistical analyses

Statistical comparisons were made using one‐way ANOVA followed by the Tukey–Kramer test for multiple comparisons (*P* < 0.05). Student's *t*‐test was used for comparisons between two groups (*P* < 0.05 and 0.01). All means are presented with their SE. The comparison of CPM for transcripts between tips of cluster‐bearing lateral roots and mature cluster roots was made using *q*‐value (*P* < 0.05) and fold‐change in edgeR. Additionally, the Welch *t*‐test was used for statistical comparison (*P* < 0.05).

## Results

### Natural and experimental growth of *Hakea laurina*

**Fig. 1:**
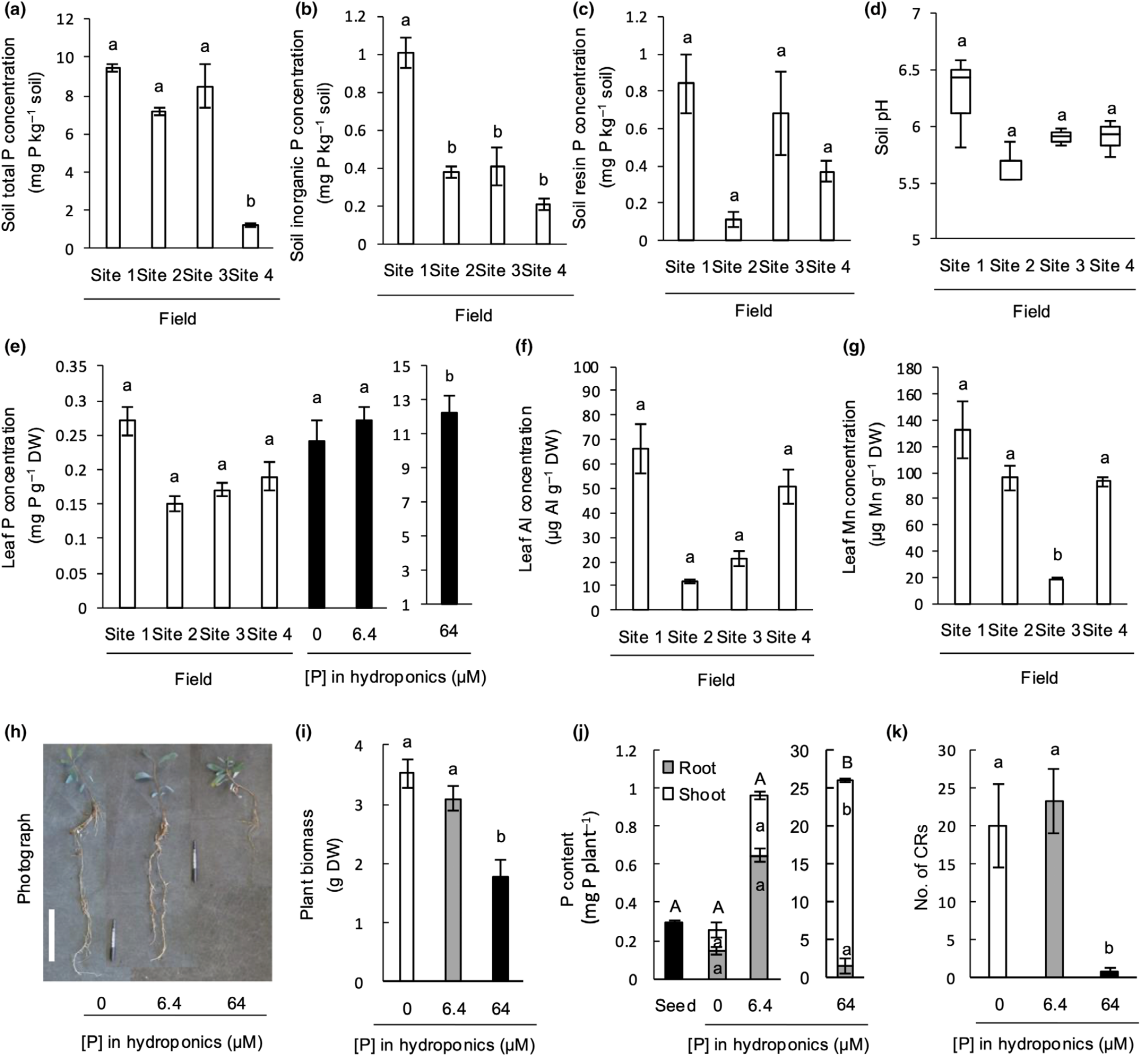
Soil and plant conditions in the natural habitat of *Hakea laurina*, and plant growth conditions in hydroponic cultivation: (a) soil total phosphorus (P) concentration, (b) soil inorganic P concentration, (c) soil resin P concentration, and (d) soil pH where *H. laurina* naturally occurs in Fitzgerald River National Park, Australia. (e) P concentration in mature leaves of *H. laurina* growing in its natural habitat or in hydroponics. (f, g) The concentration of aluminum (Al) and manganese (Mn) in mature leaves of *H. laurina* grown in its natural habitat. Coordinates of each site are given in the legend to Supporting Information Methods S1. The seedlings were hydroponically cultivated under 0, 6.4, or 64 μM of NaH_2_PO_4_ for 43 d. (h) Photograph of representative seedlings, (i) total biomass including shoot and root, (j) P content of the seedlings as well as seeds of *H. laurina*, and (k) the number of cluster roots (CRs) formed by *H. laurina* under three P treatments. Bar, 150 mm in (h). The soil total P concentration, soil resin P concentration, soil pH, leaf Al concentration, and leaf Mn concentration across four field sites, as well as plant biomass, P content, and the number of cluster roots among three P treatments, were subjected to a Tukey–Kramer test. The comparison of leaf P concentration was tested using the Tukey–Kramer test across seven samples, each with three replicates, including data from the field (*n* = 12; four field sites with three individuals each) and the glasshouse (*n* = 9; three treatments with three individuals each). Different letters indicate statistically significant differences (*P* < 0.05; Tukey–Kramer, *n* = 3). Error bars indicate SE.

We investigated growth conditions of *H. laurina* in its natural habitat to establish a benchmark for its cultivation in the laboratory. *Hakea laurina* naturally occurs in an extremely P‐impoverished environment, with soil P concentrations ranging from 1.2 ± 0.1 to 9.4 ± 0.2 mg total P kg^−1^ soil. Of this, 0.21 ± 0.03 to 1.01 ± 0.08 mg P kg^−1^ soil was inorganic P, and 0.11 ± 0.04 to 0.84 ± 0.16 mg P kg^−1^ soil was resin P across four sites in Fitzgerald River National Park, Western Australia (Fig. 1a–c). Soil pH was moderately low, ranging from 5.6 ± 0.1 to 6.0 ± 0.2 (Fig. 1d), which is not considered to cause Al toxicity. The plants at all four sites were large and healthy in appearance. The P concentration in mature leaves of *H. laurina* collected at the four sites in Fitzgerald River National Park ranged from 0.15 ± 0.01 to 0.27 ± 0.02 mg P g^−1^ DW (Fig. 1e). The concentration of Al and Mn in mature leaves was 12 ± 1 to 66 ± 10 μg Al g^−1^ DW and 19 ± 1 to 133 ± 22 μg Mn g^−1^ DW, respectively (Fig. 1f,g).

*Hakea laurina* seedlings were hydroponically cultivated under three P conditions to investigate the effect of P availability on plant biomass as well as on physiological traits. There was no significant difference in the dry mass of seedlings supplied with 0 and 6.4 μM P (Fig. 1h,i). The leaf P concentrations at 0 and 6.4 μM P supply were similar to those of the samples collected in the field, so these treatments were ecologically relevant (Fig. 1e). However, the seedlings supplied with 64 μM P for 43 d were significantly smaller than those supplied with 0 and 6.4 μM P and had much less root biomass (Fig. 1h,i). We observed a very high leaf P concentration of 12.2 ± 1.0 mg P g^−1^ DW in the 64 μM P treatment (Fig. 1e) with 94% of the P allocated to the shoot (Fig. 1j). The 0 and 6.4 μM P treatments stimulated the development of a similar number of cluster roots (Fig. 1k). Analysis of plans in their natural habitat and grown in hydroponics revealed that *H. laurina* survived in extremely P‐limited environments, whereas an excess P supply (64 μM) was toxic.

### Root exudation of carboxylates and acid phosphatase

**Fig. 2:**
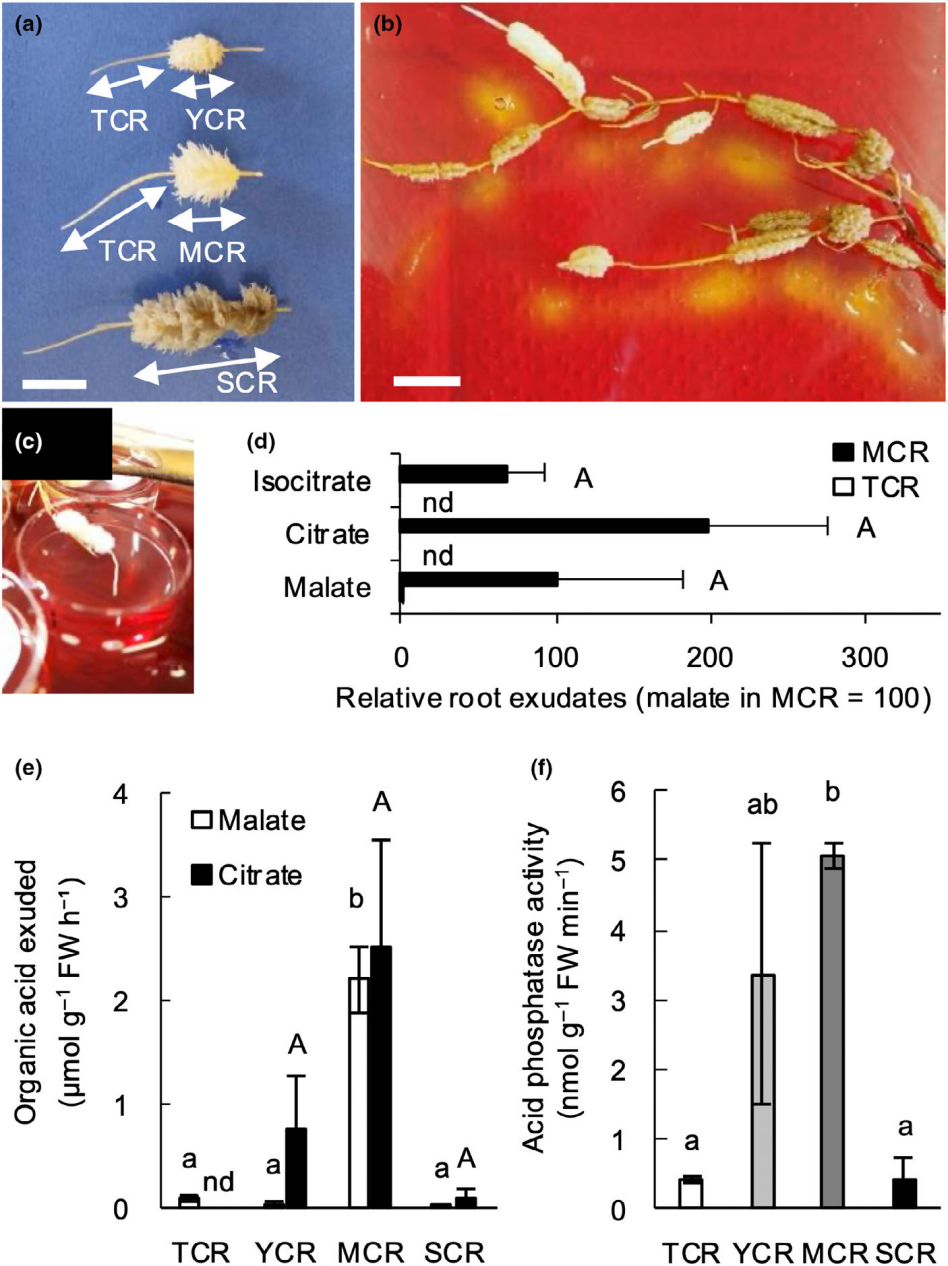
Photographs and physiological traits of cluster roots (CRs) of *Hakea laurina*. (a) Roots were divided into four developmental stages, including tips of cluster‐bearing lateral roots (TCR), young CR (YCR), mature CR (MCR) with fully elongated rootlets, and senesced CR (SCR) turning dark brown. (b) Roots were incubated for 30 min on agar containing 0.008% (w/v) bromocresol purple (pH indicator) to detect proton release and predict physiologically active CRs. (c) Traps for collecting root exudates at each developmental stage of CRs. Bars, 10 mm (a, b). (d) Three dominant organic anions in root exudates based on capillary‐electrophoresis time‐of‐flight/mass spectrometry (CE‐TOF/MS) analysis. The relative carboxylate concentration in root exudates was calculated as the concentration of malate in MCR exudates as standard (standard value = 100). Different letters indicate statistically significant differences (*P* < 0.05; nd, not detected; Tukey–Kramer, *n* = 3) among carboxylates. Error bars indicate SE. (e) Exudation rates of malate and citrate at four developmental stages of CRs. (f) Acid phosphatase activities in root exudates at four developmental stages of CRs. Relative amounts of root exudates were compared using the Tukey–Kramer test. The comparisons of exuded carboxylates and acid phosphatase activity were also tested using the Tukey–Kramer test at four developmental stages of CRs. Different letters indicate statistically significant differences (*P* < 0.05; nd, not detected; Tukey–Kramer, *n* = 3). Error bars indicate SE.

Roots were separated into four developmental stages: TCR), YCR), MCR, and SCR (Fig. 2a). Agar containing a pH indicator changed color from purple to light yellow when mature cluster roots and young cluster roots were placed on the gel, whereas agar in contact with tips of cluster‐bearing lateral roots or senescent cluster roots changed color only slightly (Fig. 2b). This color change indicated that the root segments, particularly the young and mature cluster roots, were exuding protons, which drive the exudation of carboxylates (Neumann *et al*., 2000; Shane & Lambers, 2005). To determine the carboxylate‐exudation rate and whether acid phosphatases were also released, we collected and measured exudates from cluster roots of these four root stages (Fig. 2c). Citrate, malate, and isocitrate were the three dominant carboxylates released from mature cluster roots of *H. laurina*, based on the CE‐TOF/MS analysis (Fig. 2d). We also detected lactate, *cis*‐ and *trans*‐aconitate (Fig. S1). We quantitatively determined the exudation rates of malate and citrate at each cluster‐root developmental stage. Both citrate and malate exudation peaked in mature cluster roots, averaging 2.2 ± 0.3 and 2.5 ± 1.0 μmol g^−1^ FW h^−1^, respectively (Fig. 2e). Acid phosphatase activity was also detected in the root exudates and was highest in the exudates from mature cluster roots (Fig. 2f).

### Biological pathways with increased expression in mature cluster roots and tips of cluster‐bearing lateral roots

Total RNA was successfully extracted from roots using a modified protocol for woody plant tissues based on the RNeasy Plant Mini Kit (Qiagen), as described in the Materials and Methods section. This method enables the comparison of gene expression in *H. laurina* cluster roots through *de novo* RNA‐Seq analysis. Given that the soil in the natural habitat of this species exhibited extremely low P concentrations (Fig. 1a–c), we expected that this species would express clear P‐acquisition strategies in its cluster roots. The leaf P concentration of plants, cultured hydroponically with limited P and used for RNA‐Seq analysis, was low at 0.14 ± 0.00 mg P g^−1^ DW similar to the values of plants in their natural habitat, indicating that the samples used for RNA‐Seq were ecologically relevant.

**Fig. 3:**
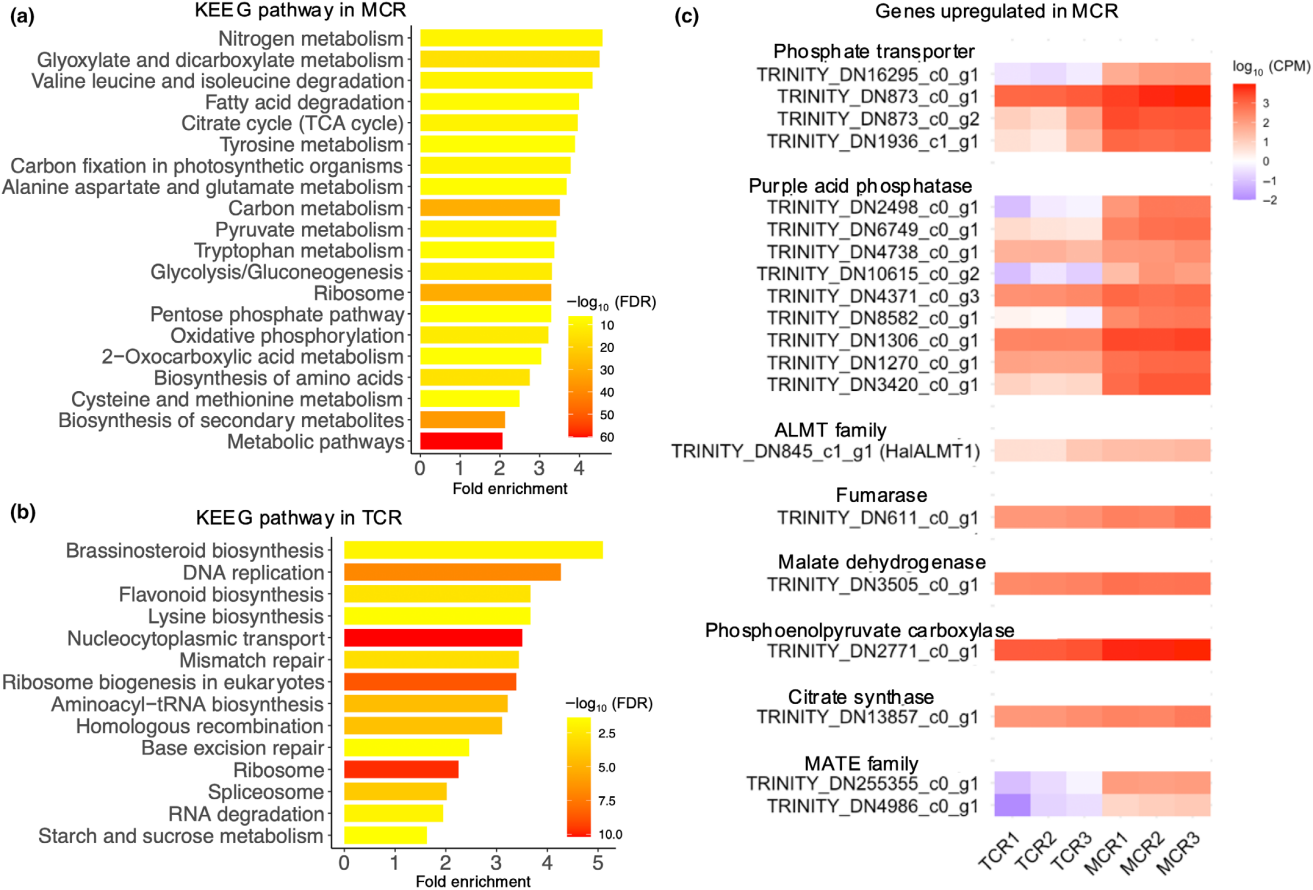
Transcriptomic analysis in *Hakea laurina* roots by RNA‐Seq. (a, b) 4210 differentially expressed genes (DEGs) upregulated in mature cluster root (MCR) and 3609 DEGs upregulated in tips of cluster‐bearing lateral roots (TCR) were subjected to Kyoto Encyclopedia of Genes and Genomes (KEGG) pathway analysis with a false discovery rate (FDR) cutoff of 0.05. The first 20 KEGG pathways with the highest fold enrichment are presented. Only 14 pathways were detected in TCR. Scale bar represents the −log_10_FDR. (c) Genes related to phosphorus (P) acquisition such as phosphate transporter, purple acid phosphatase, aluminum‐activated malate transporter family, malate dehydrogenase, phospho*enol*pyruvate carboxylase, citrate synthase, and multidrug and toxic compound extrusion family were selected based on the Welch *t*‐test (*P* < 0.05, *n* = 3) applying 4210 DEGs upregulated in MCR. Heatmap visualizing the expression of these genes in TCR and MCR. Scale bar represents the log_10_CPM. These genes are considered candidate genes for phosphorus acquisition that function in MCR. ALMT, aluminum‐activated malate transporter; MATE, multidrug and toxic compound extrusion.

RNA‐Seq analysis of mature cluster roots and tips of cluster‐bearing lateral roots revealed 183 999 contigs. After filtering out low‐signal contigs and annotating the dataset, we detected 9219 differentially expressed contigs representing 7819 DEGs with annotations between these two root types (Fig. S2; Table S3). Among these, 4210 DEGs were upregulated in mature cluster roots compared with tips of cluster‐bearing lateral roots, while the other 3609 DEGs were upregulated in tips of cluster‐bearing lateral roots compared with mature cluster roots. GO and KEGG pathway analysis using these DEGs highlighted multiple carboxylate‐related processes in mature cluster roots (Fig. 3; Table S4). For example, the KEGG pathways with the top 20 highest fold enrichments included glyoxylate and dicarboxylate metabolism in second place, citrate cycle (tricarboxylic acid (TCA) cycle) in fifth place, and carbon fixation in photosynthetic organisms in seventh place (Fig. 3a). Additionally, pathways involved in acetyl‐CoA production were included, such as valine, leucine and isoleucine degradation, fatty acid degradation, pyruvate metabolism, glycolysis, and pentose phosphate pathway (Fig. 3a). Nitrogen metabolism was the most‐enriched pathway (Fig. 3a) primarily involved in nitrogen assimilation leading to L‐glutamate synthesis. Other nitrogen‐related pathways, including tyrosine metabolism, alanine, aspartate, and glutamate metabolism and biosynthesis of amino acids were enriched in the genes upregulated in mature cluster roots (Fig. 3a). Carbon metabolism and gluconeogenesis (Fig. 3a) were also included. On the other hand, none of these pathways were significantly enriched among the DEGs upregulated in tips of cluster‐bearing lateral roots (Fig. 3b; Table S4). Most of the pathways enriched in tips of cluster‐bearing lateral roots were involved in meristem homeostasis and root growth, such as DNA replication, lysine biosynthesis, nucleocytoplasmic transport, mismatch repair, ribosome biogenesis in eukaryotes, aminoacyl‐tRNA biosynthesis, homologous recombination, spliceosome, base excision repair, and ribosome (Fig. 3b).

### Phosphorus‐starvation‐induced genes in mature cluster roots

The 4210 DEGs upregulated in mature cluster roots were numerous candidates related to P acquisition. Using a Welch *t*‐test (*P* < 0.05) on the DEGs allowed further narrowing down of candidates. The selected candidates included DEGs encoding four phosphate transporters, nine purple acid phosphatases, one ALMT family gene, one fumarase, one malate dehydrogenase, one phospho*enol*pyruvate carboxylase, one citrate synthase, and two multidrug and toxic compound extrusion (MATE) family genes (Fig. 3c).

For further investigation to identify a gene that affects carboxylate exudation, one specific transcript of interest in regulating carboxylate exudation was TRINITY_DN845_c1_g1, a DEG encoding an ALMT that is a candidate for a malate transporter. We named this transcript *HalALMT1*. The other two candidates that may be involved in citrate exudation were two MATE family transporters. However, LaMATE, which is upregulated under low‐P conditions and strongly expressed in mature cluster roots of *L. albus*, is not involved in citrate transport but in isoflavonoid transport (Uhde‐Stone *et al*., 2005; Zhou *et al*., 2021). Since several ALMTs, such as LaALMT1, AtALMT3, and GmALMT5, have been characterized as malate transporters involved in P acquisition (Peng *et al*., 2018; Maruyama *et al*., 2019; Zhou *et al*., 2020), we further investigated whether HalALMT1 mediates malate release.

The coding region of *HalALMT1* (accession no. LC833900) was 1485 bp. The deduced amino acid sequence consisted of 495 residues with a predicted molecular mass of 54.7 kDa. The predicted protein was hydrophobic having six putative transmembrane domains based on TMHMM (https://services.healthtech.dtu.dk/services/TMHMM‐2.0/), suggesting it is a membrane‐bound protein. A search of the protein database (the National Center for Biotechnology Information, https://www.ncbi.nlm.nih.gov/) identified a gene encoding ALMT10 in *Telopea speciosissima* (Proteaceae) with 78% of amino acid sequence identity. According to quantitative polymerase chain reaction analysis, *HalALMT1* mRNA accumulation in mature cluster roots was approximately eight times greater than that in tips of cluster‐bearing lateral roots (Fig. S3a).

### HalALMT1 transport activity and response to aluminum

The relative expression of *HalALMT1* tended to be upregulated after exposure to 100 μM Al^3+^ for 3 h, although this induction was not statistically significant (Fig. S3a). Within *c*. 3 kbp upstream of the *HalALMT1* coding region (accession no. LC833901), we found a total of 37 putative ART1 binding *cis*‐elements known to play a role in the Al response (GGN(T/g/a/C)V(C/A/g)S(C/G); Yokosho *et al*., 2016). However, the 15 bp STOP1 binding *cis*‐element known to meditate aluminum‐induced expression of *ALMT1* in *A. thaliana* (GGGGAGGGCTTAACT; Tokizawa *et al*., 2021) was not found in that region. We conducted a phylogenetic analysis of HalALMT1 based on predicted full‐length amino acid sequences and several well‐characterized orthologs such as 14 ALMTs from *A. thaliana*, two ALMTs from *L. albus*, and other related ALMTs to predict the element‐responsive specificity of HalALMT1 (Fig. S3b). The HalALMT1 sequence grouped in Clade 4 (Fig. S3b), displaying 53% of amino acid identity with AtALMT10 from *A. thaliana*, a sequence that remains uncharacterized, and 50.7% of amino acid identity with LaALMT1 from *L. albus* (Zhou *et al*., 2020).

**Fig. 4:**
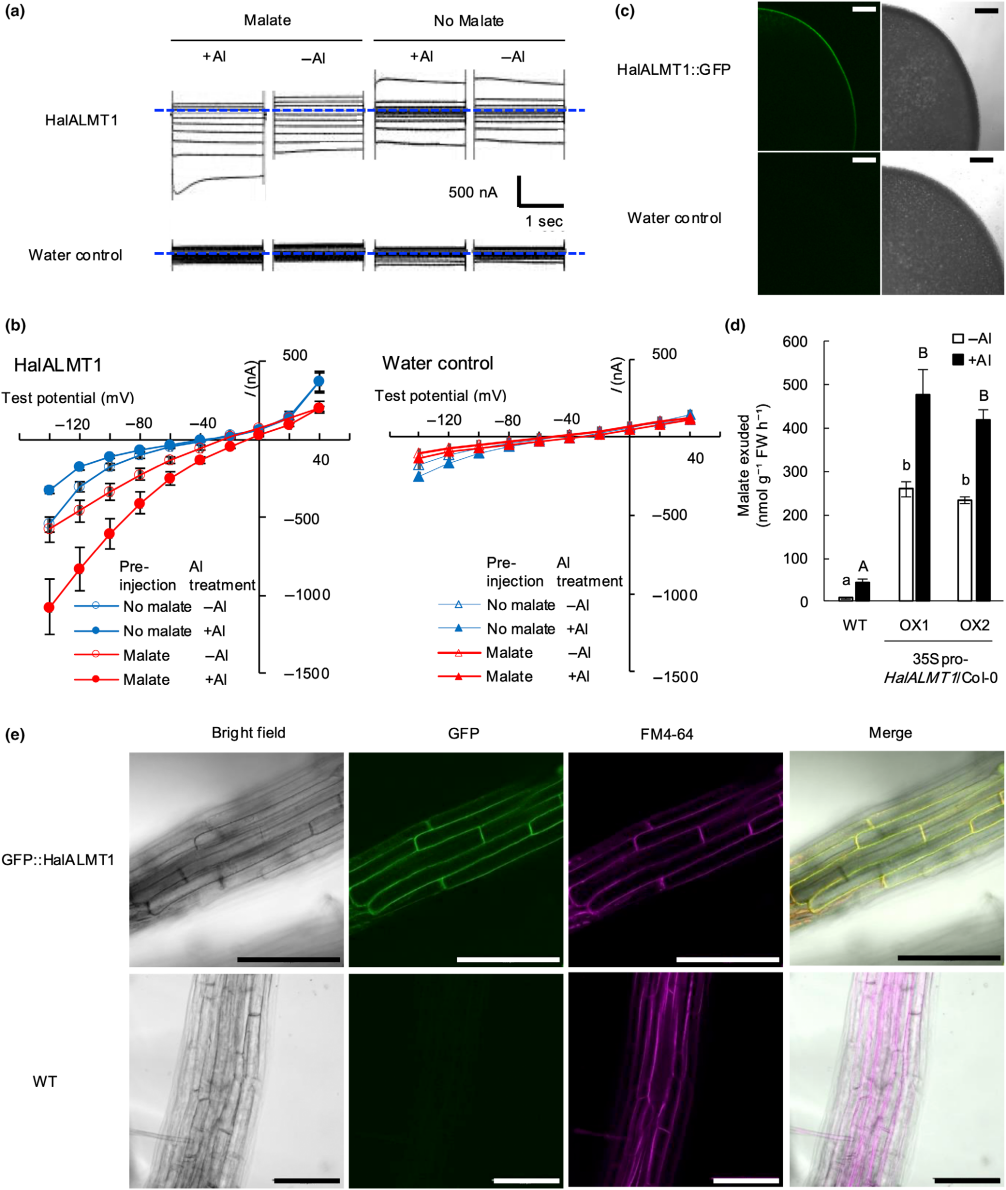
Malate‐transport capacity of Aluminum‐activated Malate Transporter 1 in *Hakea laurina* (HalALMT1). (a) Electrophysiological properties of HalALMT1 expressed in *Xenopus* oocytes. Representative currents were recorded from oocytes expressing HalALMT1 in the ND96 solution (pH 4.5) (96 mM NaCl, 1.8 mM CaCl_2_, 1 mM KCl, 0.1 mM LaCl_3_, 210 mOsm kg^−1^ with sorbitol). Water‐injected oocytes instead of cRNA‐injected oocytes were used as the control. Oocytes were pre‐injected with sodium malate before the start of measurement (Malate) or were not pre‐injected (No malate). The dotted blue line represents zero current (0 nA). (b) The oocytes were incubated for 3 to 4 d after cRNA injection, then used for the two‐electrode voltage clamp analysis after sodium malate was pre‐injected (Malate), or directly (No malate) before measurements. The oocyte membrane was clamped to the holding potentials (−20 mV), and voltage pulses were applied in 20 mV increments from −140 to +40 mV. The bath solutions were basically ND 96 pH 4.5. AlCl_3_ was added at 100 μM final concentration into the bath solution. Data are presented as the means ± SE (*n* = 12–14) and report the mean value of currents in oocytes independently obtained from two donor frogs (*n* = 14: ALMT1 pre‐injected malate and + Al, *n* = 12: others). Statistical tests were conducted at −100 mV and are shown in Supporting Information Fig. S4. (c) Localization of 35Spro‐HalALMT1::GFP protein (C‐terminal fusion) in oocytes. (d) Malate exuded (nmol g^−1^FW h^−1^) from the roots of wild‐type (WT; Col‐0) and 35Spro‐*HalALMT1*/Col‐0 (OX1 and OX2). Roots of seedlings cultivated in ½ Murashige and Skoog (MS) for 25 d were incubated in 10 ml water or 13.6 μM AlCl_3_ solution for 24 h to collect root exudates. Malate was measured by ion chromatography. Malate exudation was compared using the Tukey–Kramer test among the WT and two transgenic lines. Different letters show a statistically significant difference (*P* < 0.05; Tukey–Kramer). Error bars indicate SE. (e) Intracellular localization of 35Spro‐GFP::HalALMT1 protein (N‐terminal fusion) in transgenic seedlings. Seedlings grown under ½ MS medium for 20 d were used: bright‐field images, GFP fluorescence images, plasma membranes stained with FM4‐64, and merged images under GFP::HalALMT1 line and WT, respectively. Bars, 100 μm in all images. Al, aluminum, GFP, green fluorescent protein.

We examined the malate‐transport activity of HalALMT1 electrophysiologically in *Xenopus laevis* oocytes using the two‐electrode voltage clamp method. Oocytes expressing HalALMT1 and pre‐injected with malate exhibited substantially greater inward currents in response to a series of voltage steps from −140 to +40 mV than oocytes injected with water (Fig. 4a,b). Moreover, exposure of HalALMT1‐expressing oocytes to 100 μM AlCl_3_ (pH 4.5) further increased these currents (Figs. 4a,b, S4). Comparatively, the reversal potential, which is the voltage at which the net current is zero and indicates that HalALMT1 currents are mediated by anion efflux, shifted more in the positive direction with malate pre‐injection than that of oocytes with water pre‐injection (Fig. 4b). This shift revealed that HalALMT1 was capable of mediating malate efflux from the cell and that this process was enhanced by Al^3+^. On the other hand, pre‐injection of HalALMT1‐expressing oocytes with citrate failed to alter either inward currents or shift the reversal potential (Fig. S5). The observation of fluorescence from HalALMT1 fused with green fluorescent protein (HalALMT1::GFP) confirmed its localization to the oocyte plasma membrane (Fig. 4c).

To further investigate whether *HalALMT1* is involved in malate exudation in plants, we constructed two independent *Arabidopsis* T_2_ generation lines overexpressing *HalALMT1* driven by the Cauliflower Mosaic Virus 35S promoter (OX1 and OX2) (Fig. S6). Malate was exuded more rapidly at 246 nmol g^−1^ FW h^−1^, in the OX1 and OX2 transgenic lines than in the WT (Fig. 4d). Furthermore, the exposure of the two transgenic lines to 13.6 μM AlCl_3_ further enhanced malate exudation to an average of 448 nmol g^−1^ FW h^−1^, while the WT released malate at 11 and 43 nmol g^−1^ FW h^−1^ under −Al and +Al conditions, respectively. This confirms the responsiveness of HalALMT1 to Al^3+^.

We examined the impact of HalALMT1 expression on plant functioning during long‐term Al^3+^ exposure in two independent *Arabidopsis* T_2_ generation lines overexpressing *HalALMT1* in an *atalmt1* background. Assays by quantitative polymerase chain reaction confirmed induction of *AtALMT1* in roots of WT under +Al conditions and showed *HalALMT1* mRNA accumulation in the roots of the two independent 35Spro‐*HalALMT1*/*atalmt1* lines (Fig. S7a). The average relative root length under +Al conditions for the two complemented lines was 63% of the root length in the absence of Al^3+^, significantly greater than that of *atalmt1* (Fig. S7b,c).

Subcellular localization of HalALMT1 was examined by creating an N‐terminal fusion of its cDNA with the GFP gene and overexpressing the construct in *A. thaliana* under the control of the 35S promoter (Fig. 4e). The GFP::HalALMT1 fluorescence corresponded precisely with the red fluorescence of FM4‐64 (magenta in the images), which stained the plasma membrane in the short term (Fig. 4e).

### Exudative potential of cluster roots

**Fig. 5:**
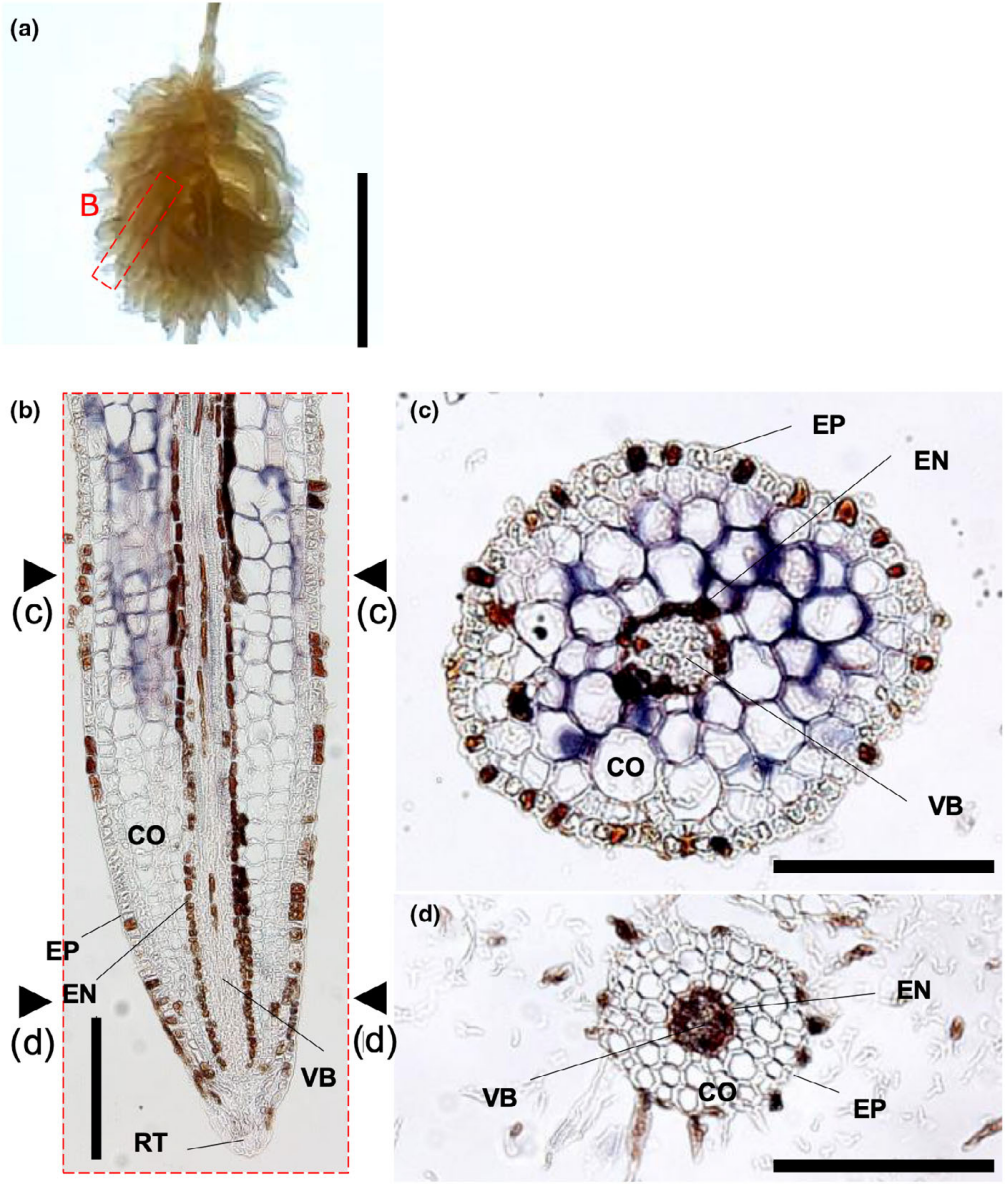
Spatial expression patterns of *Hakea laurina Aluminum‐activated Malate Transporter 1* (*HalALMT1*) mRNA in cluster rootlets of *Hakea laurina* were observed by *in situ* hybridization. Hybridization was visualized by the purple color produced with digoxigenin. (a) Cluster roots were harvested from hydroponically cultivated *H. laurina* under low‐phosphorus conditions. Bar, 0.5 cm (a). (b) A representative longitudinal section, and (c, d) cross section of a rootlet. The reproducibility of *in situ* hybridization was confirmed in at least six samples. Other observations are presented in Supporting Information Fig. S8. CO, cortex; EN, endodermis; EP, epidermis; RT, root tip; VB, vascular bundle. Bars, 200 μm (b–d).

**Fig. 6:**
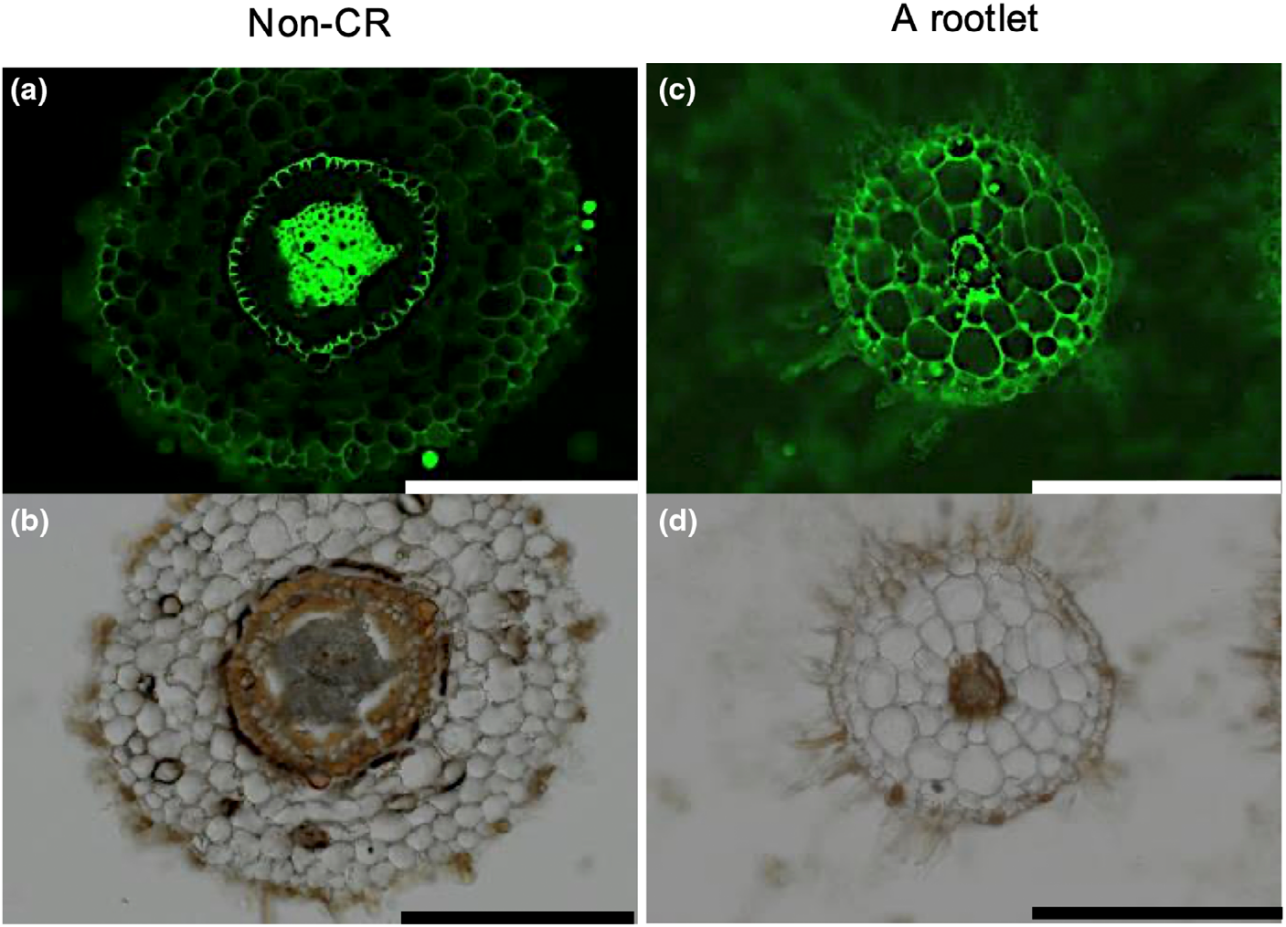
Histochemical activity of acid phosphatase for a noncluster root (non‐CR) and a cluster rootlet (a rootlet) of *Hakea laurina* using ELF97 phosphate as a substrate after *c*. 5 yr of cultivation in low‐phosphorus hydroponics. After mounting mature CRs in optimal cutting temperature compound, each 50 μm section was cut with a cryostat. The activity staining and autofluorescence on the cross section are shown in (a) and (c), and in (b) and (d), respectively. Bars, 200 μm in all images.

We determined the spatial expression patterns of *HalALMT1* mRNA in cluster roots of *H. laurina* by whole‐mount *in situ* hybridization in longitudinal and cross sections of the rootlets. The leaf P concentration of harvested seedlings was 0.16 ± 0.01 mg P g^−1^ DW, similar to that of plants in their natural habitat. *HalALMT1* mRNA accumulated in mature cortex cells *c*. 500 μm behind the rootlet tips (Figs. 5, S8). We observed fluorescence from the precipitate generated from ELF‐97 by acid phosphatase activity in specific areas of the root. In noncluster roots, fluorescence was localized around the stele, endodermis, and epidermis (Fig. 6a,b). By contrast, we observed stronger and more generalized fluorescence throughout cluster rootlets, particularly in the apoplast of the cortex where HalALMT1 was highly expressed (Fig. 6c,d). The fluorescence in noncluster roots was much weaker (Fig. 6).

**Fig. 7:**
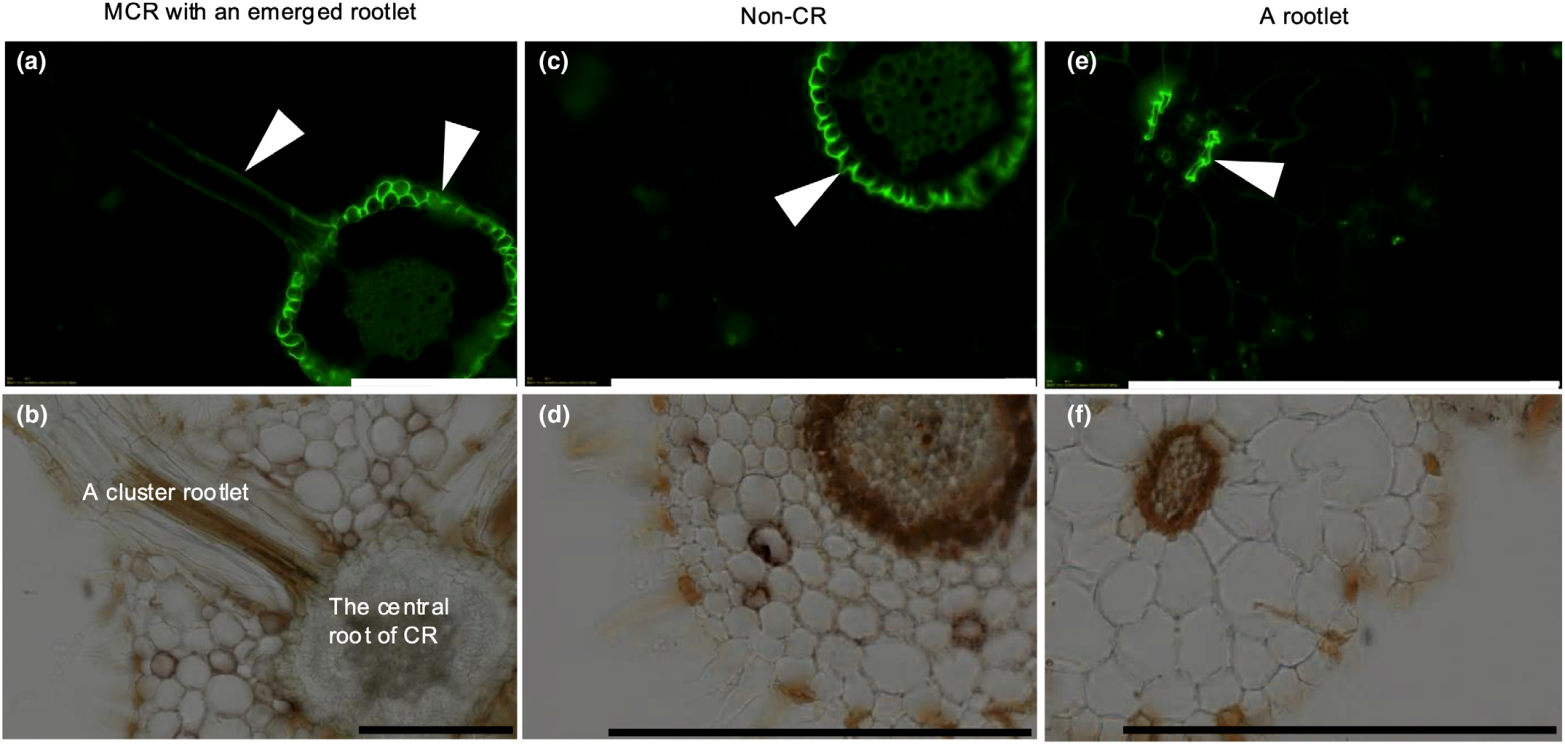
Histochemical root analysis of *Hakea laurina* after *c*. 5 yr of cultivation in hydroponics. After mounting mature cluster roots (MCR) and noncluster roots (non‐CR) in optimal cutting temperature compound, each 50‐μm section was cut with a cryostat. Cross sections were stained with 0.01% (w/v) Fluorol Yellow 088, and then, the presence of suberin was detected by yellow‐green fluorescence under a fluorescence microscope, Apex view APX100. (a) and (b) show MCR with an emerged rootlet, (c) and (d) show a mature non‐CR, (e) and (f) show a mature cluster rootlet. Arrow heads indicate suberized lamellae. Bars, 200 μm in all images.

We stained suberized cells in roots with Fluorol Yellow 088. Fluorescent cells were mainly localized to the endodermis of noncluster roots as well as the central root of cluster roots (Fig. 7a–d). We also detected partially stained cells in the endodermis of cluster rootlets, although the signal was weaker than that in the central part of the cluster roots (Fig. 7a,b,e,f). By contrast, we did not detect any suberized lamellae in exodermal cell layers of cluster rootlets (Fig. 7).

## Discussion

### *Hakea laurina* exhibited high phosphorus‐acquisition capacity in mature cluster roots

Based on our investigations in the natural habitat of *H. laurina* and the hydroponic experiment, we conclude that *H. laurina* has outstanding tolerance of low‐P conditions. The resin P concentration in the soils across the Jurien Bay, Guilderton, Yalogorup, and Warren chronosequences of coastal sand dunes in southwest Australia, known for their extreme P impoverishment, is below 2.5 mg P kg^−1^ soil (Turner *et al*., 2018). The resin P concentration in the natural habitat of *H. laurina* at Fitzgerald River National Park, which also consists of highly weathered sands, was at the lower end of those concentrations.

The physiological mechanisms of cluster roots that contribute to low‐P tolerance in *H. laurina* share similar characteristics with those of cluster roots of other Proteaceae as well as *L. albus* (Neumann *et al*., 2000; Lambers *et al*., 2022). Malate, citrate, and acid phosphatase exudation occurred in a development‐dependent manner, peaking in mature cluster roots before declining in senescing cluster roots, similar to the response in many other Proteaceae (Lambers *et al*., 2022). While different Proteaceae exhibit variation in the types of carboxylates they secrete, *H. laurina* primarily secreted citrate and malate, similar to *H. prostrata* (Shane *et al*., 2004a). *Hakea laurina* possessed a similar capacity for carboxylate secretion (2.2 μmol citrate g^−1^ FW h^−1^ and 2.5 μmol malate g^−1^ FW h^−1^) as *H. prostrata* and *L. albus*. The maximum combined secretion rate of citrate and malate in *H. prostrata* across the whole root system is 2.2 μmol g^−1^ FW h^−1^ (Shane *et al*., 2004a), while the maximum citrate secretion rate in mature cluster roots of *L. albus* is 2.4 μmol g^−1^ FW h^−1^ (Keerthisinghe *et al*., 1998).

Transcriptomic analysis identified several factors underlying enhanced carboxylate exudation and cluster‐root formation. Most of the KEGG pathways enriched in mature cluster roots are involved in carboxylate metabolism, which would support an increase in the supply of malate and citrate. The significant amounts of acid phosphatase in mature cluster roots suggests it makes an effective contribution to phosphate mobilization from organic P. The combination of organic anions and acid phosphatase synergistically enhances the mobilization of soil organic P (Furutani *et al*., 2017; Giles *et al*., 2017). Finally, the increased expression of phosphate transporters provides a mechanism for phosphate uptake.

### *Hakea laurina* sustained low internal phosphorus concentrations

*Hakea laurina* showed another low‐P tolerance strategy by maintaining plant growth at low plant internal P. The average leaf P concentration in several Proteaceae growing in their natural habitat of P‐impoverished Bassendean sand in Alison Bird Reserve near Perth is 0.26 mg P g^−1^ DW (Liu *et al*., 2023) and in Mt. Lesueur National Park near Jurien Bay is 0.27 to 0.34 mg P g^−1^ DW (Pereira *et al*., 2019). Leaf P concentration in *H. prostrata* was 0.20 mg P g^−1^ DW in the Bassendean sand of Mt. Lesueur National Park (Yan *et al*., 2019), similar to the leaf P concentration of *H. laurina* in the present study. Conversely, a high P supply suppressed *H. laurina* growth, demonstrating that it is P sensitive, like many other Proteaceae native to southwest Australia or South Africa (Shane *et al*., 2004b; Hawkins *et al*., 2008). In cluster‐root‐forming *H. prostrata*, another Proteaceae native to southwest Australia, cultivation in hydroponic solutions with a P supply of more than 50 μM P induced P toxicity, resulting in a high leaf P concentration of 9.6 mg P g^−1^ DW (Shane *et al*., 2004b). When supplied with 100 μM P, its leaf P concentration is 13.6 mg P g^−1^ DW. By contrast, *Triticum aestivum* and *Medicago truncatula* reach maximum growth without exhibiting toxicity at a leaf P concentration of 8.7 and 12.9 mg P g^−1^ DW, respectively, when supplied with 1000 μM P (Shane *et al*., 2004b). These results suggest that P excess is not common for Proteaceae native to southwest Australia. In fact, the downregulation of phosphate transporter genes under conditions of excess P supply is attenuated in *H. prostrata* compared with that in other species (Bird *et al*., 2024b), which is a primary reason for P toxicity in this species. *Hakea laurina* potentially possesses not only a great ability for P mining but also a remarkable capability to survive with extremely low internal P concentration, but it has a low tolerance for excessive P.

### Candidate genes involved in phosphorus acquisition and uptake of other elements in mature cluster roots

RNA‐Seq comparison between tips of cluster‐bearing lateral roots and mature cluster roots revealed multiple key candidate genes functioning in mature cluster roots that increase P acquisition, such as phosphate transporters, malate and citrate transporters, citrate synthases, and acid phosphatases. The accumulation of these transcripts potentially enables effective P acquisition by cluster roots. Interestingly, the top two dominant phosphate transporters, TRINITY_DN873_c0_g1 and TRINITY_DN873_c0_g2, were localized to a Proteaceae‐specific clade of PHT1 sequences that contains the *H. prostrata* sequences HpPHT1;11 and HpPHT1;12 (Nestor *et al*., 2024), with *HpPHT1;12* being induced in mature cluster roots compared with white roots in *H. prostrata* (Bird *et al*., 2024b). These results indicate that *Hakea* species upregulate Proteaceae‐specific phosphate transporters in mature cluster roots. The genes encoding the HpPHT1;11 and HpPHT1;12 sequences are a highly duplicated and rearranged array of PHT1 genes and pseudogenes within a 150 kbp segment on chromosome 11 of *H. prostrata* that is also highly rearranged in the genome of *Telopea speciosissima* (Proteaceae) compared with that of *Macadamia integrifolia* (Proteaceae) (Nestor *et al*., 2024). Genome sequencing may aid in more completely detecting PHT1 genes in *H. laurina*, as TRINITY_DN873_c0_g1 had nine isoforms based on the transcriptome development in the present study.

In addition to P‐related genes, several genes related to uptake of other elements were highly expressed in mature cluster roots, such as two nitrate‐uptake transporters (TRINITY_DN21564_c0_g1 and TRINITY_DN4631_c0_g1), three potassium channels (TRINITY_DN35317_c0_g1, TRINITY_DN5232_c0_g1, and TRINITY_DN5449_c0_g1), one zinc‐regulated transporter (ZRT)/iron‐regulated transporter (IRT)‐like protein (TRINITY_DN2164_c1_g1), two ferric chelate reductase (FER)‐like regulators of iron uptake (TRINITY_DN772_c0_g3 and TRINITY_DN4354_c1_g1), and one natural resistance‐associated macrophage protein (NRAMP) metal ion transporter (TRINITY_DN3374_c0_g1). The upregulation of the expression of these genes suggests that cluster‐root function in the uptake of elements other than P. Overall, this transcriptomic regulation appears to be a common strategy not only for P uptake but also uptake of other elements, as evidenced by *L. albus* accumulating similar transcripts in cluster roots (Secco *et al*., 2014; Le Thanh *et al*., 2021). Since *L. albus* is a crop Fabaceae and *H. laurina* is a woody Proteaceae, this strategy appears to represent convergent evolution of cluster roots.

### Functions supporting cluster‐root formation in tips of cluster‐bearing lateral roots

The fresh mass of a single mature cluster root formed by *H. prostrata* is *c*. 2.5 g, which is about five times greater than that of an emerging cluster root (Shane *et al*., 2004a). Cluster‐root‐forming species must allocate resources to cluster roots while growing under low‐P conditions. RNA‐Seq results showed 10 DEGs encoding cellulose synthase were upregulated in tips of cluster‐bearing lateral roots. Additionally, enzymes related to lignin synthesis, such as 4‐coumarate ligase, cinnamoyl‐CoA reductase, and cinnamyl alcohol dehydrogenase, were upregulated in tips of cluster‐bearing lateral roots, while none of these functions were found among the DEGs upregulated in mature cluster roots. These enzymes are involved in the formation of the plant carbon skeleton, suggesting that tips of cluster‐bearing lateral roots prioritize maturing instead of releasing carboxylates, whereas mature cluster roots are specialized in carboxylate exudation.

The enrichment in tips of cluster‐bearing lateral roots of the KEGG pathways brassinosteroid biosynthesis and flavonoid biosynthesis suggests a role in the formation of young roots, including young cluster roots. Brassinosteroids interact with other signaling molecules such as auxin or glucose and regulate lateral root development in *A. thaliana* (Bao *et al*., 2004; Gupta *et al*., 2015) in an ethylene‐independent manner (Singh *et al*., 2015). These hormones were tested in *L. albus*, where they influence cluster‐root formation (Wang *et al*., 2015). Specifically, treatment with the brassinosteroid antagonist brassinazole inhibits cluster‐root formation in hydroponic culture lacking P (Wang *et al*., 2015). Flavonoids are also involved in cluster‐root formation (Xiong *et al*., 2022). These findings suggest that the mechanisms underlying cluster‐root formation in *H. laurina* are similar to those in *L. albus*, although further studies are needed to corroborate this.

### HalALMT1‐mediated malate release under low‐phosphorus as well as aluminum stress conditions

HalALMT1 shared 51% sequence identity with LaALMT1 (Zhou *et al*., 2020), which functions as a malate transporter involved in P acquisition in cluster roots of *L. albus*. We identified a P1BS motif (Rubio *et al*., 2001) 980–987 bp upstream of the HalALMT1 coding region. This suggests the presence of a binding site for the PHR1 transcription factor that is a central switch in the plant P‐starvation response (Rubio *et al*., 2001).

Results from electrophysiological assays and overexpression of HalALMT1 in *A. thaliana* revealed that the malate transport activity of HalALMT1 was enhanced by AlCl_3_ exposure. Additionally, the recovery of root elongation observed in the complementation of *atalmt1* by 35Spro‐*HalALMT1* suggests a role for HalALMT1 in alleviating Al toxicity. Several ALMT genes have been isolated that show various stress responses while being involved in malate transport. These include family members that are activated by aluminum such as TaALMT1 (Sasaki *et al*., 2004) and AtALMT1 (Hoekenga *et al*., 2006) in Clade1, as well as ALMT family members that are induced by low P availability, such as GmALMT5 (Peng *et al*., 2018) and AtALMT3 (Maruyama *et al*., 2019) in Clade 2, and LaALMT1 (Zhou *et al*., 2020) localized to Clade 4 based on amino acid sequence analysis, which classifies into five clades (Sharma *et al*., 2016). Malate released into the rhizosphere driven by H^+^ export acidifies the rhizosphere, thereby increasing the solubility of Al^3+^ as a toxic substance inhibiting root elongation (Ryan *et al*., 1992). Given that the soils in the natural habitat of *H. laurina* have a moderately low pH, ranging from pH 5.6 to pH 6.0, the rhizosphere pH could become sufficiently acidic (e.g. pH < 5) near cluster roots releasing protons to enhance the Al^3+^ concentration. Thus, HalALMT1 contributes to both P acquisition and alleviation of Al toxicity.

To alleviate Al toxicity, plants usually either accumulate Al followed by sequestration with carboxylates or exclude Al by releasing carboxylates (Kochian *et al*., 2005). The Al concentration in mature leaves of *H. laurina* was relatively low, ranging from 12 to 66 μg Al g^−1^ DW. Some plants accumulate high levels of Al in their leaves, thus coping with acidic and Al‐toxic conditions, reaching over 1000 μg Al g^−1^ DW (Jansen *et al*., 2002). By contrast, Al‐excluders typically accumulate much lower amounts even in acidic environments (Osaki *et al*., 1998). Our results indicate that *H. laurina* is an Al‐excluding plant through carboxylates release, similar to many other Proteaceae (Lambers *et al*., 2022).

### Phosphorus‐acquisition strategies in the cortex of mature cluster roots

**Fig. 8:**
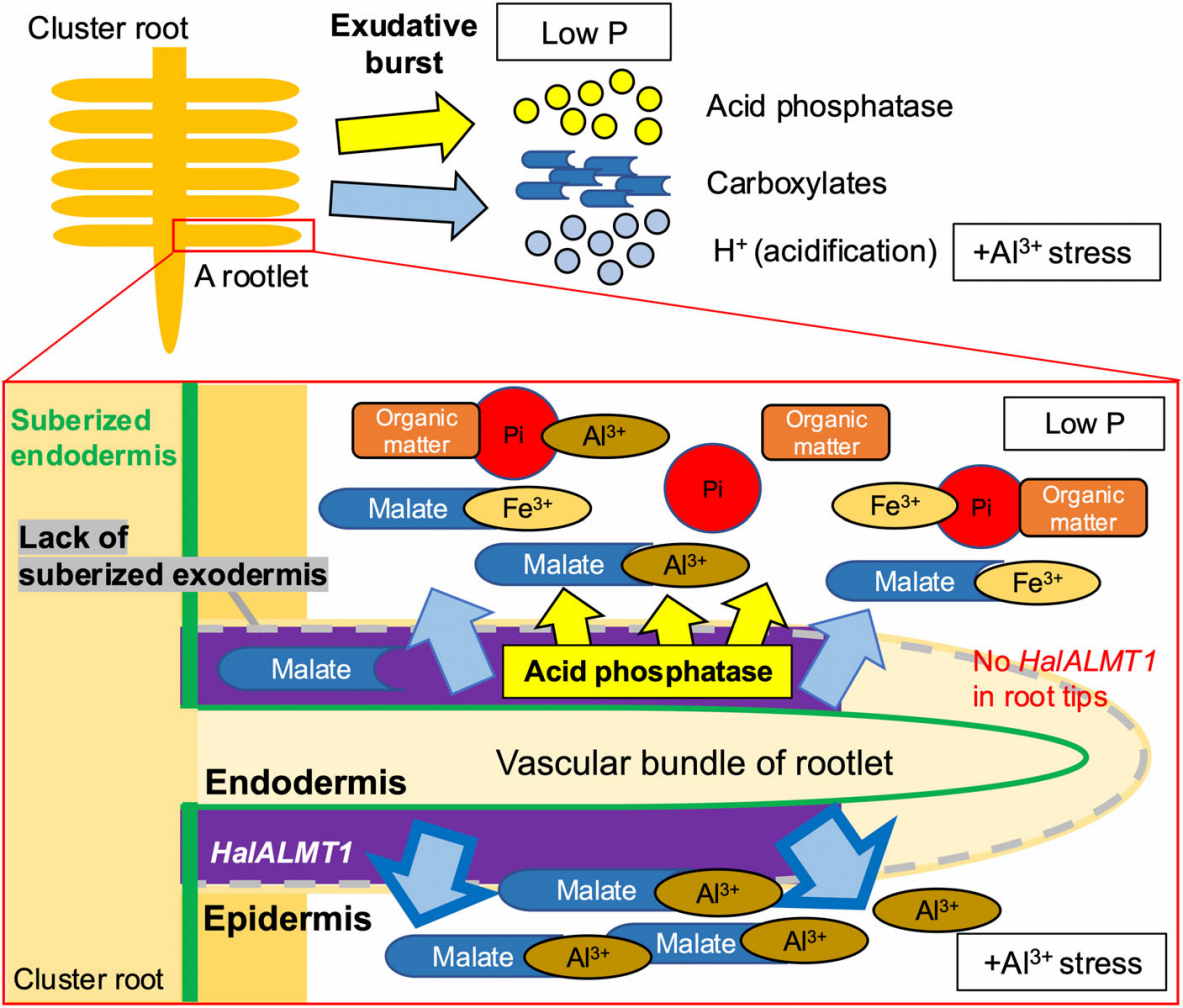
Schematic diagram illustrating the functioning of cluster roots of *Hakea laurina*. *Hakea laurina* exhibits an exudation burst of acid phosphatase and carboxylates (malate and citrate) with H^+^ release under low‐phosphorus (P) availability, which increases the Al^3+^ concentration in the soil. Aluminum‐activated Malate Transporter 1 in *Hakea laurina* (*HalALMT1*) is transcriptionally upregulated in the cortex of mature cluster rootlets, facilitating malate release under low‐P conditions. The activity of acid phosphatase was particularly strong in the apoplast of the cortex. Their malate transport activities were enhanced by exposure to Al^3+^, which may occur in acid soil. *Hakea laurina* only develops a suberized endodermis but it does not develop a suberized exodermis in cluster rootlets, which may facilitate malate exudation and acid phosphatase release from the cortex into the rhizosphere.

For the effective access to P in soil, *H. laurina* released root exudates in a cell‐dependent manner. *In situ* hybridization demonstrated that *HalALMT1* was not localized in the root tip, but in the mature cortical cells of the rootlets, which coincides with the location of enhanced acid phosphatase activity. Both exudation of malate and secretion of acid phosphatase were most pronounced in mature cluster roots among four developmental stages. Moreover, the lack of a suberized exodermis in cluster rootlets suggests that malate and acid phosphatase are exuded from the apoplast of the cortex into the rhizosphere to enhance P uptake (Fig. 8). Other ALMTs involved in P uptake localize to different cell types, for example *AtALMT3* is expressed in the root epidermis, particularly in root hair cells (Maruyama *et al*., 2019); *LaALMT1* is expressed in the stele of cluster‐rootlet apices (Zhou *et al*., 2020); and *GmALMT5* is expressed along the entire root system (Peng *et al*., 2018). The larger area expressing *HalALMT1*, from which malate is exuded, along with enhanced production of acid phosphatase, specifically in the cortex rather than the epidermis, might allow for an instantaneous burst of the exudation of malate and acid phosphatase. Proteaceae species that have been studied so far only have a suberized endodermis (Lambers *et al*., 2018). Therefore, other Proteaceae species may express similar strategies to access P under P‐limited environments.

### Conclusions

This study highlights one of the adaptations of *H. laurina* (Proteaceae) that enable it to thrive in severely P‐impoverished environments in southwest Australia. For the first time, we investigated the transcriptome of the roots of this highly P‐efficient *Hakea*, shedding light on the molecular mechanisms underlying the functioning of cluster roots, a distinctive root structure, in response to low plant‐available P. We found an up‐regulated transcript in mature cluster roots, HalALMT1, which mediated malate efflux under both low P and Al stress, to be specifically expressed in mature cortex cells of the rootlets (Fig. 8). This unique localization differs from that of previously characterized ALMTs. Our results confirm the absence of a suberized exodermis in *H. laurina* which would prevent exudate movement into the rhizosphere, and reveal high levels of acid phosphatase activity in the cortex. These findings suggest that the cortex, rather than the epidermis, of the cluster rootlets is the primary site where *Hakea* releases a larger quantity of root exudates. These insights into plant adaptations to nutrient‐poor soils may pave the way for strategies that enable crops to maintain growth with less P fertilizer.

## Competing interests

None declared.

## Author contributions

HY and JW designed the study. HY performed the experiments and collected the data. The following coauthors made significant contribution to the experiments: LRB conducted *Arabidopsis* mutant experiment. AT and SN performed RNA‐Seq analysis. TK and HM conducted the hydroponic experiment. WT performed *in situ* hybridization experiments. T, AO and KT carried out CE‐TOF/MS analysis. TW carried out ICP‐MS analysis. TS performed electrophysiological assays. STL organized the fieldwork. HY wrote the manuscript. PMF, HL, TS and JW made significant contributions to the structure of the paper and the writing. All authors contributed critically to the experiments and gave final approval for publication.

## Disclaimer

The New Phytologist Foundation remains neutral with regard to jurisdictional claims in maps and in any institutional affiliations.

## Supporting information


**Fig. S1** Relative amount of organic anions in root exudates from *Hakea laurina*.
**Fig. S2** Results of differentially expressed genes between tips of cluster‐bearing lateral roots and mature cluster roots formed by *Hakea laurina*.
**Fig. S3** Quantitative polymerase chain reaction of *Hakea laurina Aluminum‐activated Malate Transporter 1* and phylogenetic tree of aluminum‐activated malate transporter family.
**Fig. S4** Intensity of an electric current (*l*) recorded from oocytes expressing or not expressing *Hakea laurina Aluminum‐activated Malate Transporter 1*, with or without malate injection, at an applied voltage of −100 mV.
**Fig. S5** Mean current–voltage curves recorded from oocytes expressing *Hakea laurina Aluminum‐activated Malate Transporter 1*and oocytes injected with water, malate, and citrate.
**Fig. S6** Quantitative polymerase chain reaction of *Hakea laurina Aluminum‐activated Malate Transporter 1* (*HalALMT1*) in roots of Col‐0 (wild‐type) and two transgenics 35Spro‐*HalALMT1* in Col‐0 (OX1 and OX2) of *Arabidopsis thaliana*.
**Fig. S7** Effect of long‐term Al^3+^ treatment on root growth of wild‐type, *atalmt1*, and two transgenics 35Spro‐*HalALMT1* in *atalmt1*(OX1 and OX2) of *Arabidopsis thaliana*.
**Fig. S8** Other observed images of *Hakea laurina Aluminum‐activated Malate Transporter 1* mRNA in cluster rootlets of *H. laurina* using the *in situ* hybridization presented in Fig. 5.
**Methods S1** Four sampling sites for *Hakea laurina* in Fitzgerald River National Park in southwest Australia.
**Methods S2** Extraction of genes differentially expressed between tips of cluster‐bearing lateral roots and mature cluster roots formed by *Hakea laurina*.
**Methods S3** An alignment of homologous transcripts coding for aluminum‐activated malate transporters in *Hakea laurina* to design a prove fragment for *in situ* hybridization.
**Table S1** Primer sequences for quantitative polymerase chain reaction.
**Table S2** Primer sequences for cloning.

**Table S3** Counts per million of differentially expressed genes upregulated in mature cluster roots and tips of cluster‐bearing lateral roots.
**Table S4** Results of gene ontology analysis and Kyoto Encyclopedia of Genes and Genomes pathway analysis using differentially expressed genes upregulated in mature cluster roots and tips of cluster‐bearing lateral roots.
Please note: Wiley is not responsible for the content or functionality of any Supporting Information supplied by the authors. Any queries (other than missing material) should be directed to the *New Phytologist* Central Office.

## Data Availability

Sequence data of *HalALMT1* and HalALMT1 promoter can be found in the International Nucleotide Sequence Database (INSD) under the following accession nos.: LC833900 (https://www.ncbi.nlm.nih.gov/nuccore/LC833900) and LC833901 (https://www.ncbi.nlm.nih.gov/nuccore/LC833901). RNA‐Seq data generated in this study have been deposited in the NCBI GEO database with the accession no. GSE279190.
