# Supplementary material for: HalALMT1 mediates malate efflux in the cortex of mature cluster rootlets of Hakea laurina, occurring naturally in severely phosphorus‐impoverished soil
Source: New Phytol. 2025 Feb 24;246(6):2597–616. doi: 10.1111/nph.70010 (PMC12095975; doi:10.1111/nph.70010)
Supplement: Supplementary file 1 — Fig. S1 Relative amount of organic anions in root exudates from Hakea laurina. Fig. S2 Results of differentially expressed genes between tips of cluster‐bearing lateral roots and mature cluster roots formed by Hakea laurina. Fig. S3 Quantitative polymerase chain reaction of Hakea laurina Aluminum‐activated Malate Transporter 1 and phylogenetic tree of aluminum‐activated malate transporter family. Fig. S4 Intensity of an electric current (l) recorded from oocytes expressing or not expressing Hakea laurina Aluminum‐activated Malate Transporter 1, with or without malate injection, at an applied voltage of −100 mV. Fig. S5 Mean current–voltage curves recorded from oocytes expressing Hakea laurina Aluminum‐activated Malate Transporter 1and oocytes injected with water, malate, and citrate. Fig. S6 Quantitative polymerase chain reaction of Hakea laurina Aluminum‐activated Malate Transporter 1 (HalALMT1) in roots of Col‐0 (wild‐type) and two transgenics 35Spro‐HalALMT1 in Col‐0 (OX1 and OX2) of Arabidopsis thaliana. Fig. S7 Effect of long‐term Al3+ treatment on root growth of wild‐type, atalmt1, and two transgenics 35Spro‐HalALMT1 in atalmt1(OX1 and OX2) of Arabidopsis thaliana. Fig. S8 Other observed images of Hakea laurina Aluminum‐activated Malate Transporter 1 mRNA in cluster rootlets of H. laurina using the in situ hybridization presented in Fig. 5. Methods S1 Four sampling sites for Hakea laurina in Fitzgerald River National Park in southwest Australia. Methods S2 Extraction of genes differentially expressed between tips of cluster‐bearing lateral roots and mature cluster roots formed by Hakea laurina. Methods S3 An alignment of homologous transcripts coding for aluminum‐activated malate transporters in Hakea laurina to design a prove fragment for in situ hybridization. Table S1 Primer sequences for quantitative polymerase chain reaction. Table S2 Primer sequences for cloning. [file NPH-246-2597-s001.pdf]

New Phytologist Supporting Information

Article title: HalALMT1 mediates malate efflux in the cortex of mature cluster rootlets of *Hakea laurina*, occurring naturally in severely phosphorus-impoverished soil

Authors: Hirotsuna Yamada, Lydia Ratna Bunthara, Akira Tanaka, Takuro Kohama, Hayato Maruyama, Wakana Tanaka, Sho Nishida, Tantriani, Akira Oikawa, Keitaro Tawaraya, Toshihiro Watanabe, Shu Tong Liu, Patrick M. Finnegan, Hans Lambers, Takayuki Sasaki, Jun Wasaki

Article acceptance date: 03 February 2025

The following Supporting Information is available for this article:

Methods S1 Four sampling sites for *Hakea laurina* in Fitzgerald River National Park in southwest Australia.

The coordinates for site1, site2, site3, and site4 are 34°11'57.0"S 119°21'36.3"E, 34°12'47.4"S 119°26'08.9"E, 34°10'36.0"S 119°33'39.9"E, and 34°10'07.2"S 119°30'35.5"E, respectively. Australian Bureau of Statistics (Jul2021-Jun2026), Digital boundary files, ABS Website, accessed 20 August 2024.

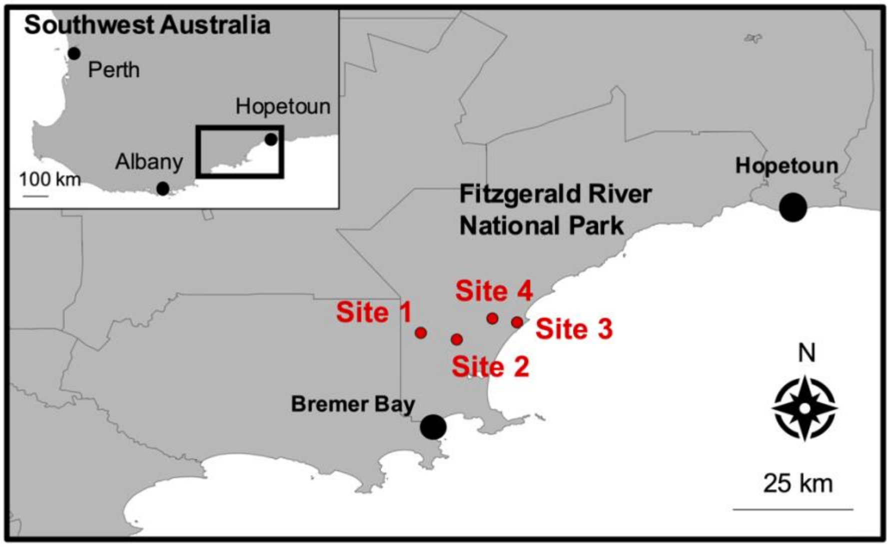

Methods S2 Extraction of genes differentially expressed between tips of cluster-bearing lateral roots (TCR) and mature cluster roots (MCR) formed by *Hakea laurina*. Total RNA extracted from roots of *Hakea laurina* hydroponically cultivated with low phosphorus (P) concentration for approximately three years was sequenced. The cDNA libraries for RNA-Seq were conducted with an NEBNext Ultra ll Directional RNA Library Prep Kit for Illumina, NEBNext Multiplex Oligos for Illumina, and NEBNext Poly(A) mRNA Magnetic Isolation Module. After being qualified and quantified by NEBNext Library Quant Kit for Illumina, the libraries were paired-end sequenced each 150 bp. Raw sequence data were cleaned up by removing the adapter sequences and low-quality sequences in Trimmomatic (version 0.39). Cleaned data were analyzed in Trinity (version 2.15.1) to assemble transcript fragments. The counts of assembled contigs were quantified using Salmon (version 1.9.0) and expressed as counts per million (CPM). They were normalized across these samples in edgeR (version 4.0.14).

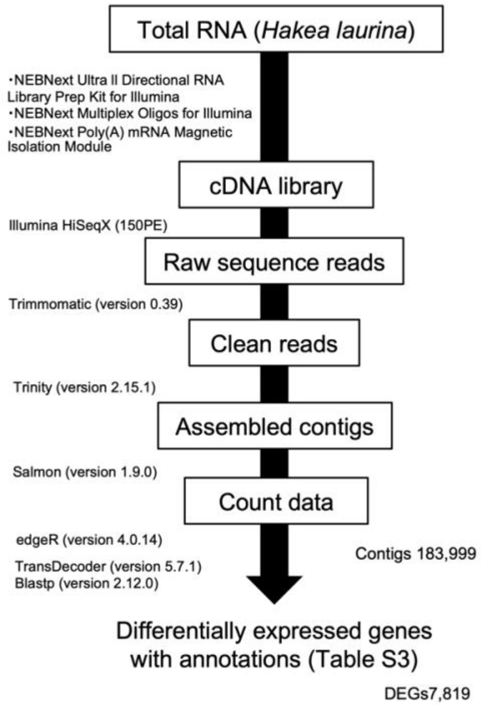

Methods S3 An alignment of homologous transcripts coding for ALMTs in *Hakea laurina* to design a prove fragment for *in situ* hybridization.

The deduced HalALMT1 amino acid sequence was subjected to Blastp (version 2.12.0) against all putative open reading frames of >100 amino acid residues to detect highly homologous proteins. The transcript of *HalALMT1* and the top five homologous transcripts with 3'-UTRs were aligned using ClustalW (Thompson et al., 1994). Arrows indicate parts of the forward and reverse primers used to create the probe fragment (Table S2). The red square box indicates the stop codon of HalALMT1.

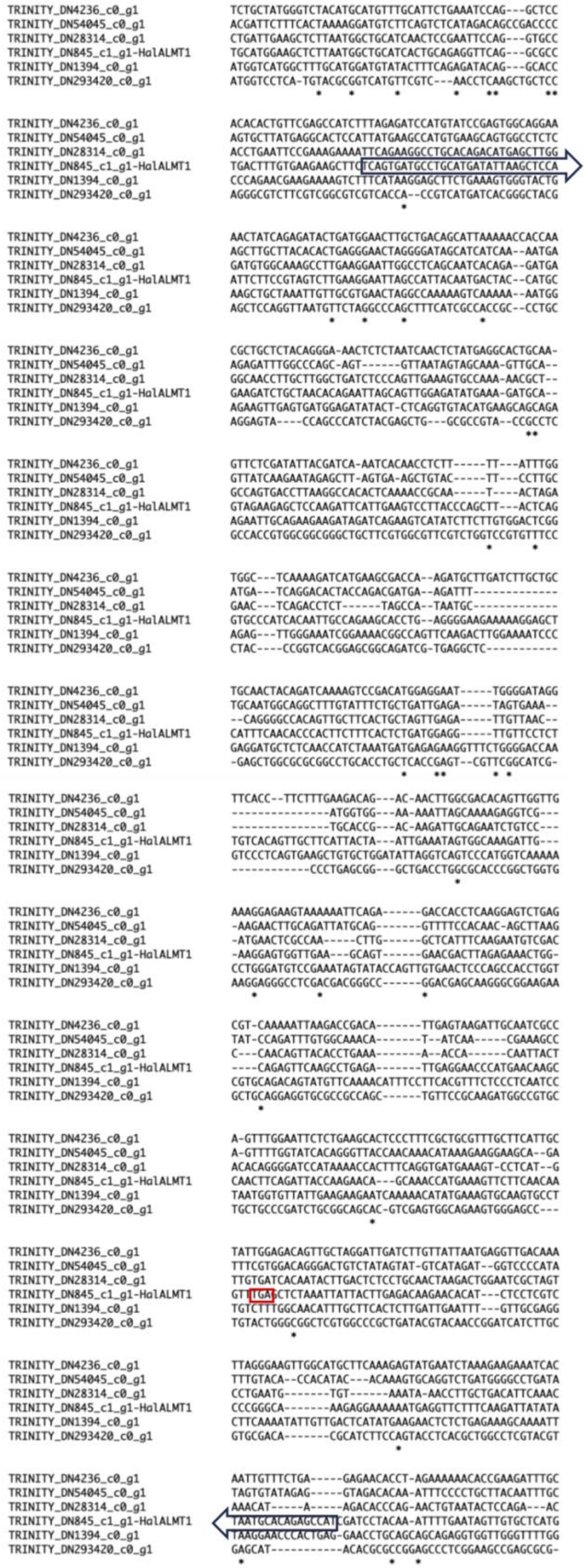

Fig. S1 The relative amount of organic anions in root exudates from *Hakea laurina*. Carboxylates were detected by CE-TOF/MS. The relative carboxylate concentration in root exudates was calculated relative to the concentration of malate in mature cluster root (MCR) (= 100). Relative amounts of root exudates were compared using the Tukey-Kramer test. Different letters indicate statistically-significant differences ($p < 0.05$; Tukey-Kramer, n = 3). Error bars indicate standard errors.

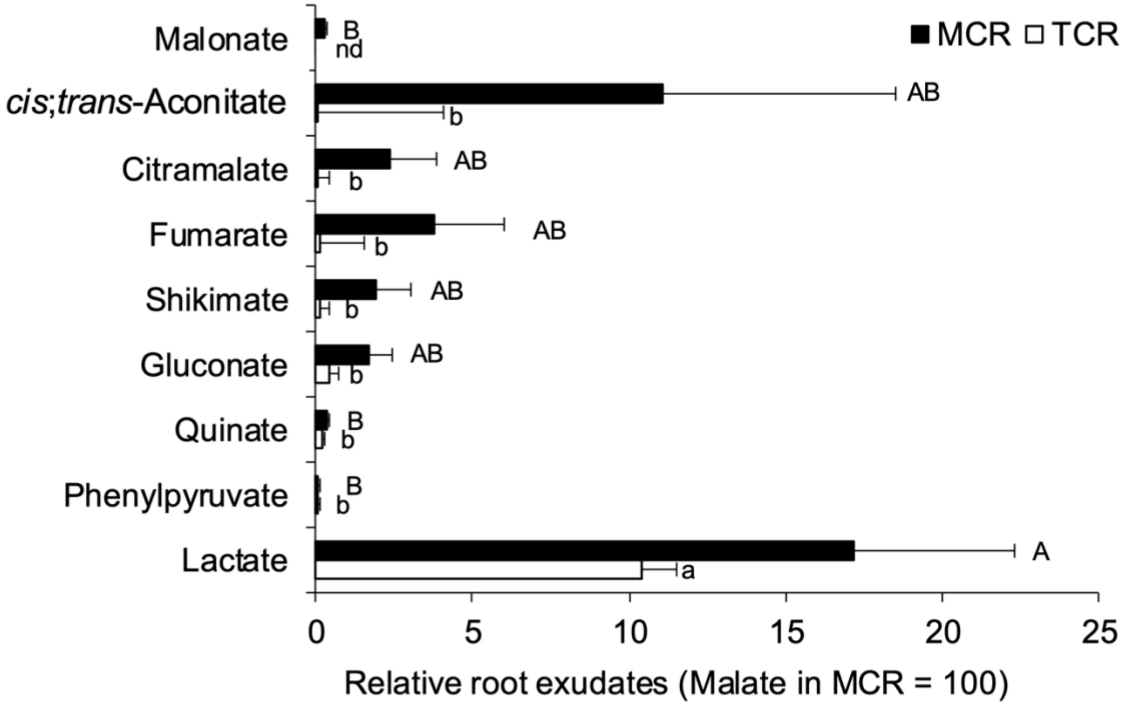

Fig. S2 Results of differentially expressed genes between tips of cluster-bearing lateral roots (TCR) and mature cluster roots (MCR) formed by *Hakea laurina*. (a) Differentially-expressed contigs with $q < 0.05$ and $|\log_2$ fold-change of (MCR/TCR)$|$ ≥ 1 were detected as differentially-expressed contigs. The x-axis (M) shows the average $\log_2$CPM, and the y-axis (A) shows the $\log_2$ fold-change gene expression. Magenta represents 9,215 significantly differently-expressed contigs. (b) Contigs with CPM below 0.4 were removed. In total, 77,014 contigs were detected. Putative open reading frames of >100 amino acid residues were searched in TransDecoder (version 5.7.1) and annotated homologs of *Arabidopsis thaliana* by Blastp (version 2.12.0). The *A. thaliana* reference proteome data were retrieved from TAIR website (https://www.arabidopsis.org/download_files/Proteins/TAIR10_protein_lists/TAIR10_pep_20110103_representative_gene_model). Finally, 7,819 annotated differentially-expressed genes (DEGs) were detected and shared in Table S3.

(a)

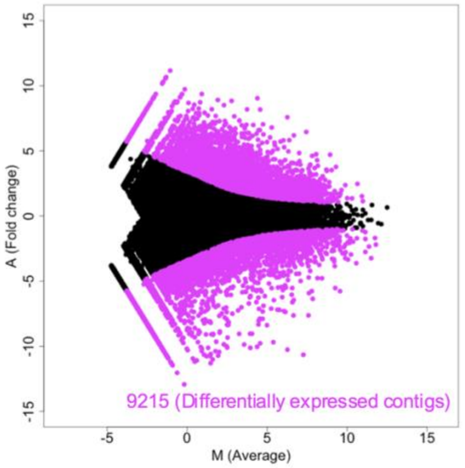

(b)

Analysis	the number of genes
CPM>0.4	77,014
Differentially expressed contigs	9,219
Annotated DEGs	7,819
DEGs upregulated in MCR	4,210
DEGs upregulated in TCR	3,609

Fig. S3 qPCR of *HalALMT1* and phylogenetic tree of ALMT family. (a) Relative *HalALMT1* expression in tips of cluster-bearing lateral roots (TCR) and mature cluster roots (MCR) of *Hakea laurina* grown without phosphorus (–P) and when exposed to $AlCl_3$ (-P+Al treatment). The expression in TCR is used as standard (= 1.0). As housekeeping gene, we selected a gene encoding an elongation factor. Relative expression was compared using the Tukey-Kramer test between TCR and MCR under -P treatment, as well as between -P and -P+Al treatments in MCR. ** indicates a statistically-significant difference ($p < 0.01$; Student's *t*-test, n = 3). Error bar indicates standard error. (b) Phylogenetic relationships among HalALMT1 found in the *Hakea laurina* and several homologs from *Arabidopsis thaliana* (At), *Brassica napus* (Bn), *Glycine max* (Gm), *Lupinus albus* (La), *Secale cereale* (Sc), *Triticum aestivum* (Ta) based on amino acid sequences. A red-circle highlighted ALMT is a target HaALMT1. Blue square-highlighted ALMTs have been characterized as Al^{3+}-activated or Al^{3+}-induced ALMTs, and green triangle-highlighted ALMTs have been reported to be involved in P uptake.

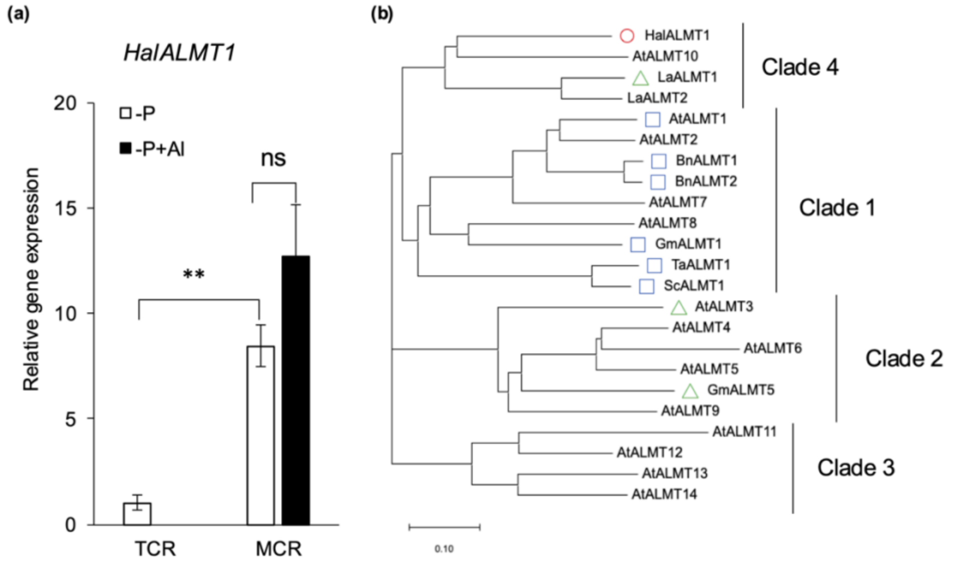

Fig. S4 The intensity of an electric current (*I*) recorded from oocytes expressing or not expressing HalATLM1, with or without malate injection, at an applied voltage of -100 mV.

These values are derived from Fig. 4b. The intensity of an electric current between -Al and +Al under HalALMT1-expressing oocytes, and between oocytes expressing or not expressing HalATLMT1 with malate induction under both -Al and +Al conditions at -100 mV was compared using Student's *t*-test (*, $p < 0.05$; **, $p < 0.01$; ns, not significant, i.e., $p > 0.05$). Data are presented as means ± standard error (n = 12-14).

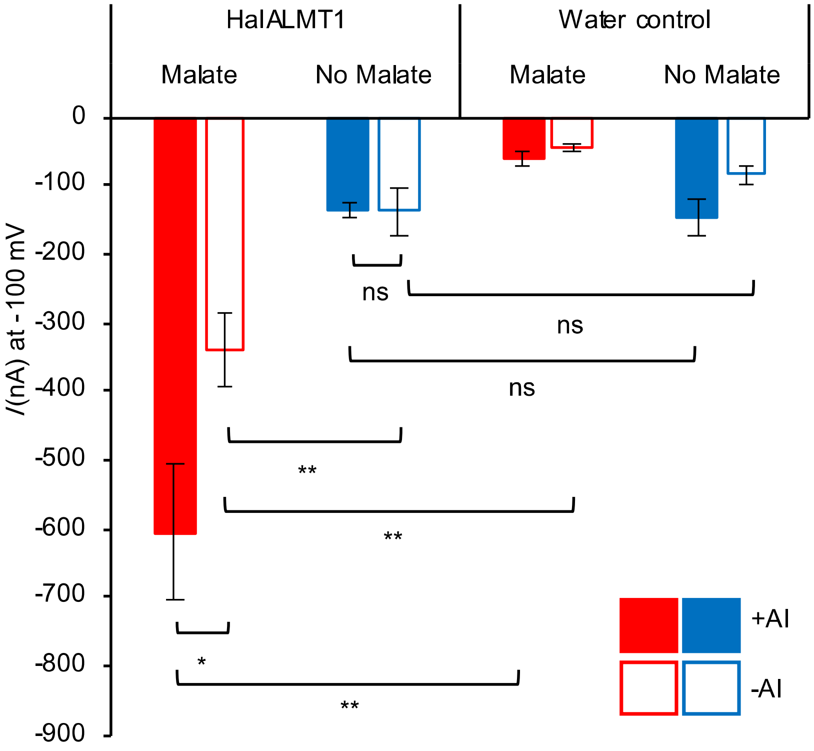

Fig. S5 Mean current-voltage curves recorded from oocytes expressing HalALMT1 and oocytes injected with water, malate, and citrate. The oocytes were used directly (no injection) for the two-electrode voltage-clamp measurements. The oocyte membrane was clamped to the holding potentials (-20 mV), and voltage pulses were applied in 20 mV increments from -140 to +40 mV. The bath solutions were ND 96 pH 4.5 [96 mM NaCl, 1.8 mM $CaCl_2$, 1 mM KCl, 0.1 mM $LaCl_3$, 210 mOsm kg^{-1} with sorbitol]. All measurements were performed with $AlCl_3$ (100 µM) in the bath solution. Data are presented as means ± standard error (n = 6-7).

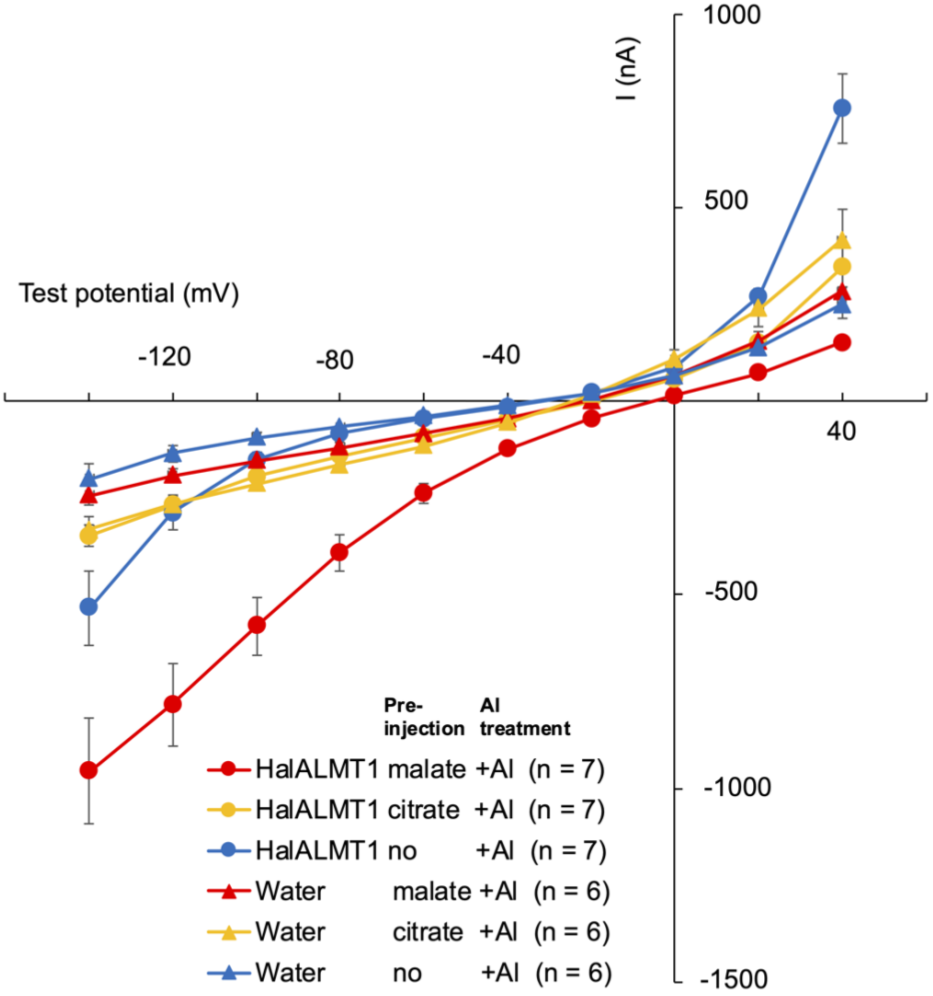

Fig. S6 qPCR of *HalALMT1* in roots of Col-0 (wildtype; WT) and two transgenics 35Spro-*HalALMT1* in Col-0 (OX1 and OX2) of *Arabidopsis thaliana*. Relative gene expression for *HalALMT1* in roots. The expression of WT is standard (=1.0). Relative expression was compared using the Student's *t*-test between transgenic lines. Different letters show a statistically-significant difference ($p < 0.05$; Student's *t*-test). Error bars indicate standard error.

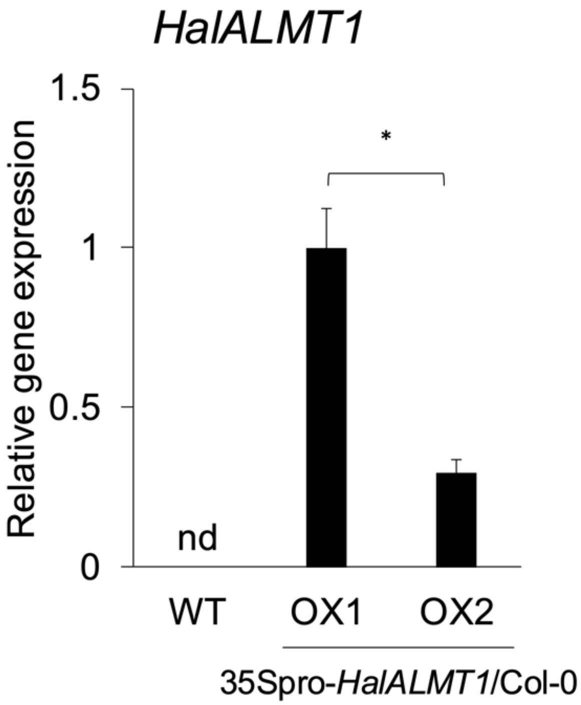

Fig. S7 The effect of long-term Al^{3+} treatment on root growth of wildtype (WT), *atalmt1*, and two transgenics 35Spro-*HalALMT1* in *atalmt1*(OX1 and OX2) of *Arabidopsis thaliana*.

Relative root length of 35Spro-*HalALMT1*/*atalmt1* under Al condition. WT, *atalmt1*, OX1, and OX2 cultivated under ½ MS medium for 10 days were transferred to ½ MS plates with or without 300 µM $AlCl_3$ (pH 4.8). The length of roots after seven days of cultivation with or without aluminum (+Al or -Al) was measured. (a) Relative gene expression for *AtALMT1* and *HalALMT1* in roots. The expression of wildtype and OX1 is standard (=1.0) for AtALMT1 and HaALMT1, respectively. Relative gene expression of *AtALMT1* and *HalALMT1* was compared using the Tukey-Kramer test among wildtype under -Al and +Al treatments, an *atalmt1* mutant, and two transgenic lines, as applicable. (b) Photograph of representatives for *atalmt1* under -Al/+Al and 35S:*HalALMT1* in *atalmt1* under -Al/+Al. (c) Relative root length under Al condition (%) was calculated as (root length under +Al)/(root length under -Al)*100. Relative root length under Al conditions was tested using the Tukey-Kramer test among wildtype, an *atalmt1* mutant, and two transgenic lines. Different letters show a statistically-significant difference ($p < 0.05$; Tukey-Kramer). Error bars indicate standard error.

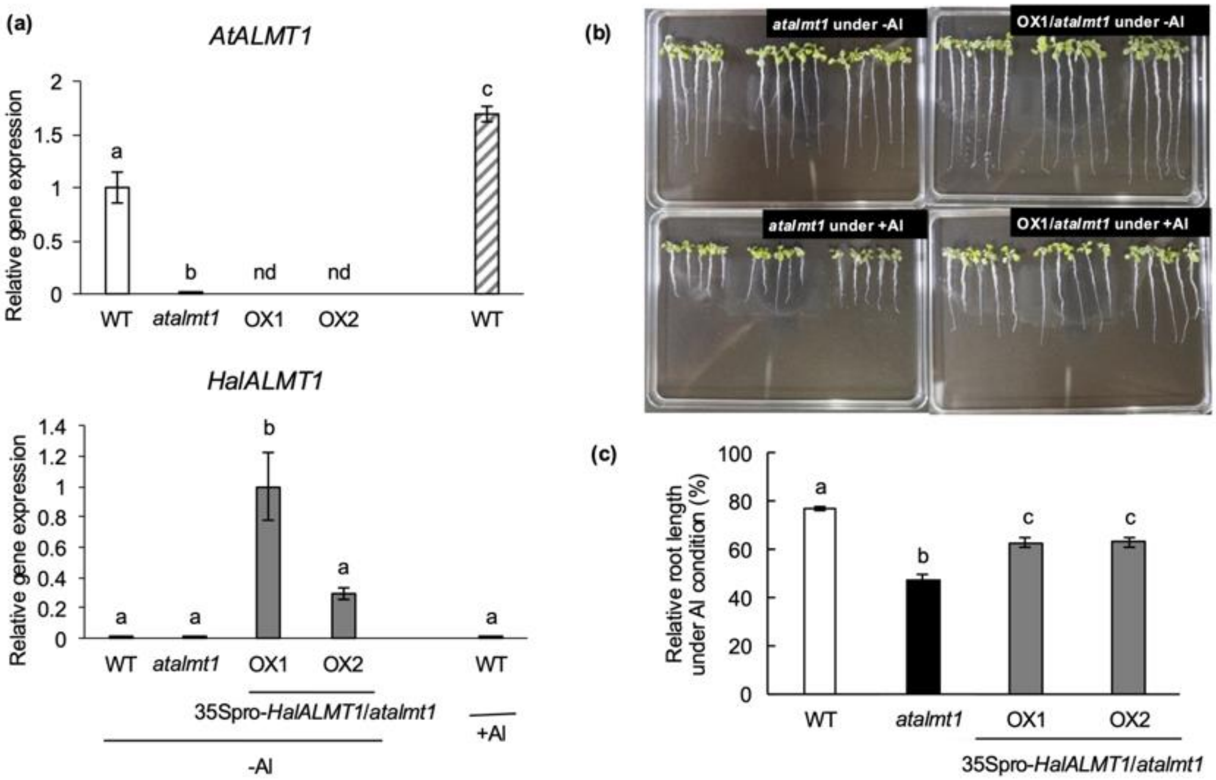

(a)
AtALMT1
Relative gene expression
2
1.5
1
0.5
0
a
b
nd
nd
c
WT
atalmt1
OX1
OX2
WT
HalALMT1
Relative gene expression
1.4
1.2
1
0.8
0.6
0.4
0.2
0
a
a
b
a
a
WT
atalmt1
OX1
OX2
WT
35Spro-HalALMT1/atalmt1
-Al
+Al
(b)
atalmt1 under -Al
OX1/atalmt1 under -Al
atalmt1 under +Al
OX1/atalmt1 under +Al
(c)
Relative root length under Al condition (%)
100
80
60
40
20
0
a
b
c
c
WT
atalmt1
OX1
OX2
35Spro-HalALMT1/atalmt1

Fig. S8 Other observed images of *HalALMT1* mRNA in cluster rootlets of *Hakea laurina* using the *in situ* hybridization presented in Fig. 5. Cluster roots were harvested from hydroponically-cultivated *Hakea laurina* under low-phosphorus conditions. (a), (b), (c), and (d) are longitudinal sections and (e) is a cross-section of a rootlet. All scale bars: 200 μm.

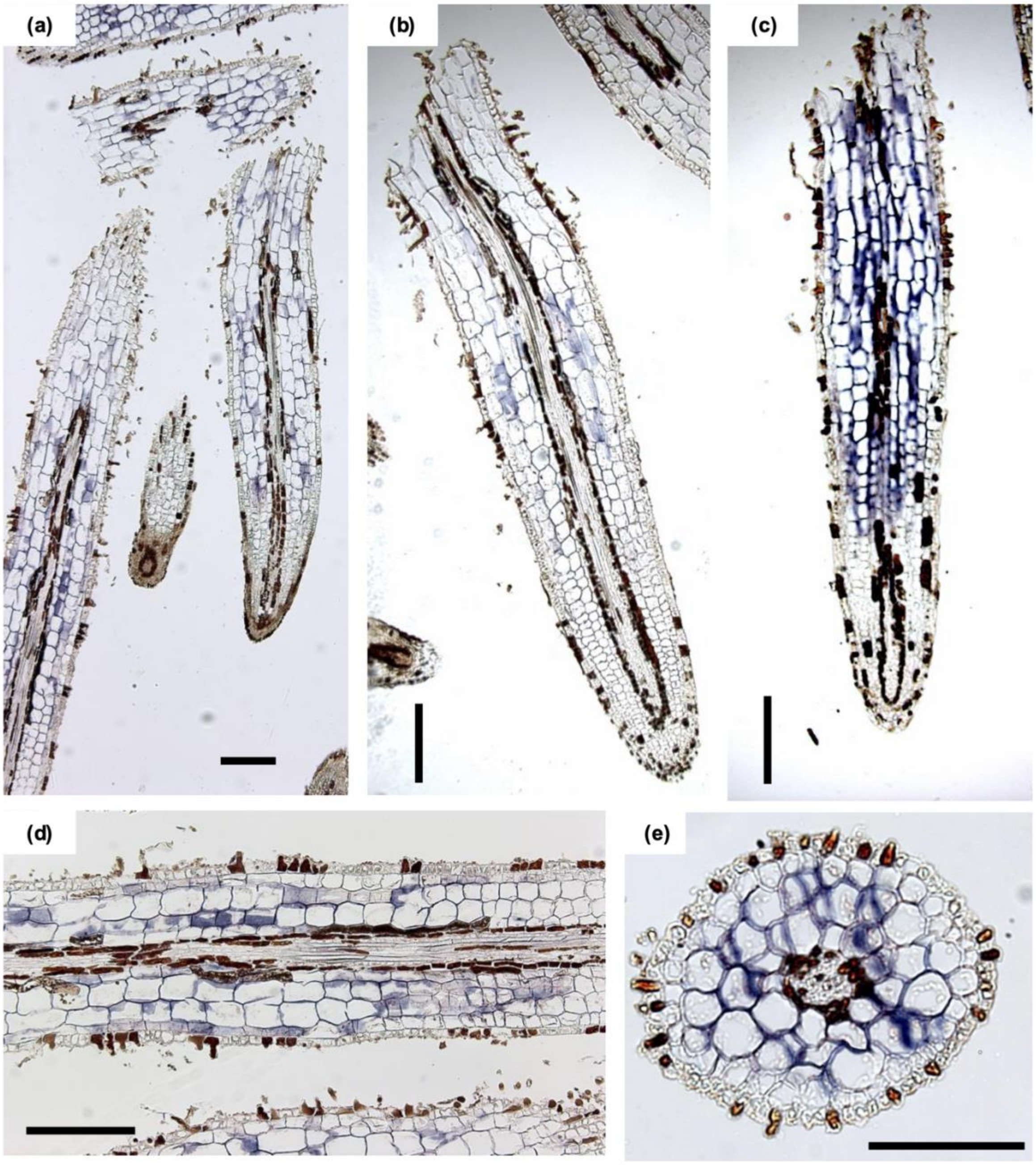

Table S1 Primer sequences for qPCR

gene	primer	Sequence (5'->3')
HalALMT1	Forward	TATTGCGTTCCACCGTACGT
(in Arabidopsis)	Reverse	TGAATCTCTTGCATCAGAGGA
HalEF1a	Forward	GTCGATTCTGGAAAGTCGACC
	Reverse	AATGTCAATGGTGATACCACGC
AtALMT1	Forward	GGCCGACCGTGCTATACGAG
	Reverse	CATGAGTCCTGTGAACTCCC
HalALMT1	Forward	ACGAACACTGATGGCAAGGT
(in Arabidopsis)	Reverse	ACACCCAACTGCAATACCCT

Table S2 Primer sequences for cloning

Name of construct	primer	Sequence (5'->3')
pXBG2	Forward	CAACTTTGGCAGATCATGGTTCATGCCAAGGAAACTT
(HalALMT1)	Reverse	AGTGGTAACCAGATCTCAAACTTGTTGAAGAACTTTCATGG
pCR^{TM}8/GW/TOPO	Forward	ATGGTTCATGCCAAGGAAACTTC
(HalALMT1-full)	Reverse	TCAAACTTGTTGAAGAACTTTCATGG
(HalALMT1 without stop codon)	Reverse	AACTTGTTGAAGAACTTTCATGGTTTG
HalALMT1 inner	Forward	CACTGGGTAGCTAACCAATC
	Reverse	ATCATGCAGGCATCACTGAG
pGWB5 -> pXBG2	Forward	CAACTTTGGCAGATCATGGTTCATGCCAAGGAAACTT
(HalALMT1::GFP)	Reverse	AGTGGTAACCAGATCTTACTTGTACAGCTCGTCCATGCCG
pGWB6 -> pXBG2	Forward	CAACTTTGGCAGATCATGGTGAGCAAGGGCGA
(GFP::HalALMT1)	Reverse	AGTGGTAACCAGATCTCAAACTTGTTGAAGAACTTTCATGG
Nested 1st PCR	Forward	CAGAATCTGGGTTCTTCCACA
	Reverse	CATTGCTTCTCTGTAGCCTCC
Nested 2nd PCR	Forward	GCCTGGCTAGGGTTCTTAGAT
	Reverse	GAAGTTTCCTTGGCATGAACC

Primer walking 1	Forward	CAGCTTGATTTCGGTATCGGG
Primer walking 2	Forward	GCTAAAACCTTCTCCCACTGC
Primer walking 3	Reverse	GTGACTATCGATCTCAGCCACA
Primer walking 4	Reverse	GAGGAATGATAGCTAAGTTTGGC
Primer walking 5	Reverse	CTTCCACTCAGAGCCACTTC
Primer walking 6	Reverse	CTTCAAAGCACTCACTCCTCAC
Promoter cloning	Forward	Same as Primer walking 2
(pHalALMT1)	Reverse	Same as Primer walking 5
Probe	Forward	CGGGCCCCCCCTCGAATGGCTCTGTGCATTATATATAATCTTG
	Reverse	TACCGTCGACCTCGATCAGTGATGCCTGCATGATATTAAG

See separate Excel file for:

Table S3 The counts per million (CPM) of differentially expressed genes (DEGs) up-regulated in mature cluster roots (MCR) and tips of cluster-bearing lateral roots (TCR) **.**

Table S4 The results of gene ontology (GO) analysis and Kyoto Encyclopedia of Genes and Genomes (KEGG) pathway analysis using differentially expressed genes (DEGs) up-regulated in mature cluster roots (MCR) and tips of cluster-bearing lateral roots (TCR).
